# Dynamic body size evolution during speciation of predatory cichlid fishes in the Lake Malawi superradiation

**DOI:** 10.1101/gr.281193.125

**Published:** 2026-05

**Authors:** Liandong Yang, Holly Scott, Benjamin P. Ngatunga, Asilatu H. Shechonge, George F. Turner, Martin J. Genner

**Affiliations:** 1 School of Biological Sciences, University of Bristol, Bristol BS8 1TQ, United Kingdom;; 2 State Key Laboratory of Breeding Biotechnology and Sustainable Aquaculture, Institute of Hydrobiology, Chinese Academy of Sciences, Wuhan 430072, China;; 3 Tanzania Fisheries Research Institute (TAFIRI), Dar es Salaam, Tanzania;; 4 School of Environmental & Natural Sciences, Bangor University, Bangor, Gwynedd LL57 2UW, United Kingdom

## Abstract

Evolutionary divergence in body size is common in animal adaptive radiations and is often associated with differences in key ecological traits, including habitat use and prey consumption. Here we characterize a notable case of body size–associated adaptive radiation in a group of predatory open water cichlid fish species from the Lake Malawi catchment. Using whole-genome sequences, we show that body size differences have evolved multiple times in the focal genus, *Rhamphochromis*, and that the group possesses well-defined signals of ancient interspecific hybridization. We identify genetic variants strongly associated with body size and show that these variants are connected to genes enriched for functions in vertebrate skeletal and nervous system development. We focus our analyses on two species of *Rhamphochromis* endemic to Lake Kingiri, a small (600 m diameter) crater lake geographically isolated from the main body of Lake Malawi but within the catchment. We show that these two ecomorphologically divergent sympatric species—one small-bodied, the other larger-bodied—share a unique common ancestor and diverged from one another ∼2000 years ago. We demonstrate strong directional selection focused on the larger-bodied Kingiri species, specifically on genetic variants connected to genes with anatomical development and nervous system function. Collectively, these results are supportive of body size–associated speciation taking place rapidly in the Lake Malawi cichlid fish superradiation. We conclude that body size–associated genetic variants have been important targets of selection during large-scale cichlid fish diversification, including in a crater lake sympatric speciation context.

Body size is intrinsically associated with multiple life history, as well as physiological and ecological traits (Peters 1983; Woodward et al. 2005) and, in fishes, is strongly linked to diet (Romanuk et al. 2011), habitat use (Clarke 2021) and migratory behavior (Burns and Bloom 2020). Furthermore, divergence in body size between sister taxa is seen in many prominent fish adaptive radiations, including three-spined sticklebacks (Smith et al. 2020), Arctic charr (Gudbrandsson et al. 2019), Alpine whitefishes (De-Kayne et al. 2022), notothenioid icefishes (Colombo et al. 2015), gobies (Troyer et al. 2025), and cichlid fishes (Gonzalez-Voyer et al. 2009; Steele and López-Fernández 2014). Therefore, body size evolution is an important component of adaptive radiation in fishes, but the genetic basis of this evolution has been explored in relatively few cases (Troyer et al. 2025). Studies exploring genetic variation associated with growth-related traits in fishes has tended to focus on species of commercial importance (Liu et al. 2016; Yoshida and Yáñez 2021; Yáñez et al. 2023), with studies on other fish species being relatively uncommon (Laine et al. 2013; Takahashi et al. 2021; DeLorenzo et al. 2023). In contrast, genetic variation underpinning body size divergence in other vertebrates has been more widely studied, and there is now good evidence supporting a polygenic basis to body size variation in humans as well as domestic mammals and birds (Sutter et al. 2007; Bouwman et al. 2018; Zhou et al. 2018).

The superradiations of cichlid fishes in the East African lakes Malawi, Victoria, and Tanganyika are among the most species rich of all vertebrate adaptive radiations. In each of these lakes, there is extreme body size divergence, and body size is at least partly linked to trophic ecology (Takahashi and Koblmüller 2011; Konings 2016), although given complex associations between body size trophic level in fishes (Keppeler et al. 2020), this requires detailed study. Within these radiations, there are cases in which sister taxa have highly divergent body sizes, suggesting body size divergence can associate with speciation. For example, the Lake Tanganyika cichlid *Telmatochromis temporalis* has undergone divergence into a larger rock-living ecotype and a smaller shell-living ecotype (Winkelmann et al. 2014). Currently, we have a very limited understanding of the genomic basis of body size divergence in cichlids (Takahashi et al. 2021; DeLorenzo et al. 2023). Nevertheless, because of their high levels of phenotypic disparity, coupled with low levels of genomic divergence (Malinsky et al. 2018), there is potential to use these radiations as models to learn more about the drivers of body size evolution in fishes.

The Lake Malawi cichlid superradiation comprises three subradiations, the Pseudotropheina, the Cyrtocarina, and the Rhamphochromina (Oliver 2024), and in each of these, there is considerable size variation associated with ecological and morphological divergence (Konings 2016). Perhaps the most remarkable interspecific size variation is within *Rhamphochromis*, a genus of predators within the Rhamphochromina, with representative adults ranging in standard length from ∼7 cm (*Rhamphochromis* sp. “kingiri dwarf,” one of the smallest species in the Malawi radiation) to >40 cm (*Rhamphochromis* sp. “nkhwazi,” the largest species in the entire radiation). Here we focus on this genus *Rhamphochromis*, which in total contains 15 species. Twelve of these species are found in the main body of Lake Malawi, and they typically have broad distributions around the lake, albeit with some species-specific habitat preferences (Turner 1996; Turner and Genner 2025). Beyond Lake Malawi itself, one very small-bodied species has been sampled only from satellite Lake Chilingali (*Rhamphochromis* sp. “chilingali”) (Genner et al. 2007) and is now thought to be extinct in the wild (Turner et al. 2023). The final two species are endemic to the small (∼600 m diameter, 34 m deep) crater Lake Kingiri (Turner et al. 2019), namely, *Rhamphochromis* sp. “kingiri large” (20 cm standard length) and *Rhamphochromis* sp. “kingiri dwarf” (7 cm standard length).

In this study, we used the large range in body size in the genus *Rhamphochromis* to explore the genetic basis of body size in cichlids using genome sequence data. We first investigated repeated body size divergence during the evolution of the genus by reconstructing the evolutionary relationships of species. We then identified genomic loci associated with body size and tested if these loci are associated with genes expressed during development. Next, we explored the evidence for body size–associated sympatric speciation in Lake Kingiri, using genome data to date the divergence event, test for gene flow with species found outside the crater lake, and identify suites of genes under selection.

## Results

### Body size variation among *Rhamphochromis* species

**Figure 1.**
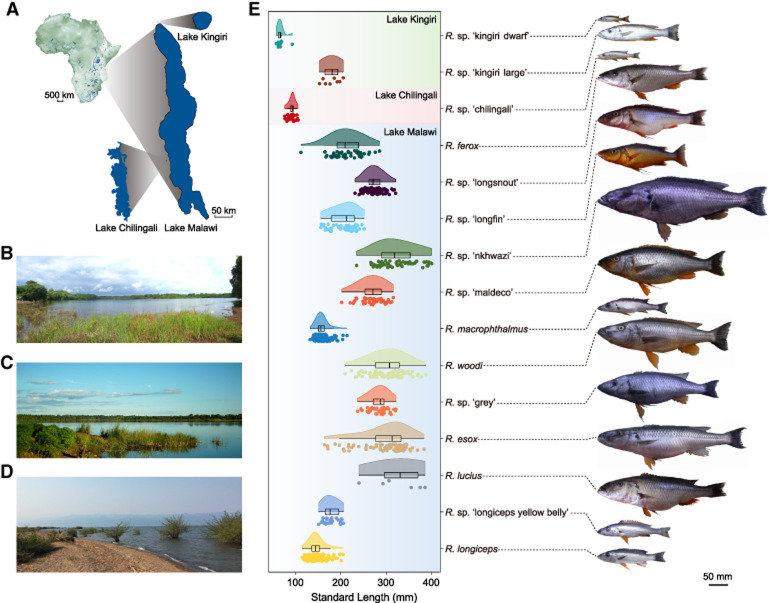
Sampling sites and body size variation in *Rhamphochromis* cichlid fish species. (*A*) Map of Lake Kingiri, Lake Chilingali, and Lake Malawi. Lake Kingiri has a diameter of ∼600 m, and Lake Chilingali has a length of ∼5.1 km. (*B*–*D*) Pictures from the shoreline of Lake Kingiri (*B*), Lake Chilingali (*C*), and Lake Malawi (*D*). (*E*) Variation of standard length and images across each of the 15 *Rhamphochromis* cichlid fish species. Relative scaling of fish pictures is to their approximate body sizes. The map of Africa is generated using data from GEBCO (https://www.gebco.net/) and Hydrolakes (https://www.hydrosheds.org/products/hydrolakes). Lake outlines redrawn from OpenStreetMap (© OpenStreetMap contributors, available under Open Database License ODbL).

From 2004 to 2011, we collected *Rhamphochromis* from three key water bodies: Lake Malawi, Lake Kingiri, and Lake Chilingali (Fig. 1). In total, we collected a total of 877 specimens representing 15 *Rhamphochromis* species that we distinguish based on body size, aspects of head, tooth, and jaw morphology, as well as male breeding colors (Supplemental Tables S1, S2). We quantified multiple morphological traits from these specimens, including dorsal spines, dorsal rays, anal rays, and standard length. Notably, standard length showed more than fourfold variation among the 15 *Rhamphochromis* cichlid fish species (Fig. 1). This exceptional disparity in body size underscores the remarkable evolutionary diversity within this group.

### Phylogeny, population structure, and evolutionary history

To resolve the evolutionary relationships among *Rhamphochromis species*, we used whole-genome sequence data from 69 individual cichlid fishes, including 67 representatives from the 15 *Rhamphochromis* species, 48 of which were newly sequenced for this study, along with two individuals from outgroup species. The average sequencing depth was 10.3×, with an average read mapping rate of 95.81% to the reference genome and a genome coverage of 97.17% (Supplemental Table S3). One sample, the only representative in our data set of *Rhamphochromis* sp. “longiceps yellow belly” (0.7×), was excluded from the whole-genome phylogenetic analysis owing to low coverage but was retained for mitochondrial genome phylogenetic analysis. In total, we identified 23,039,948 SNPs and 6,157,291 short insertions and deletions (indels). After applying stringent filtering criteria, 2,816,637 high-quality variants were retained for downstream analyses.

**Figure 2.**
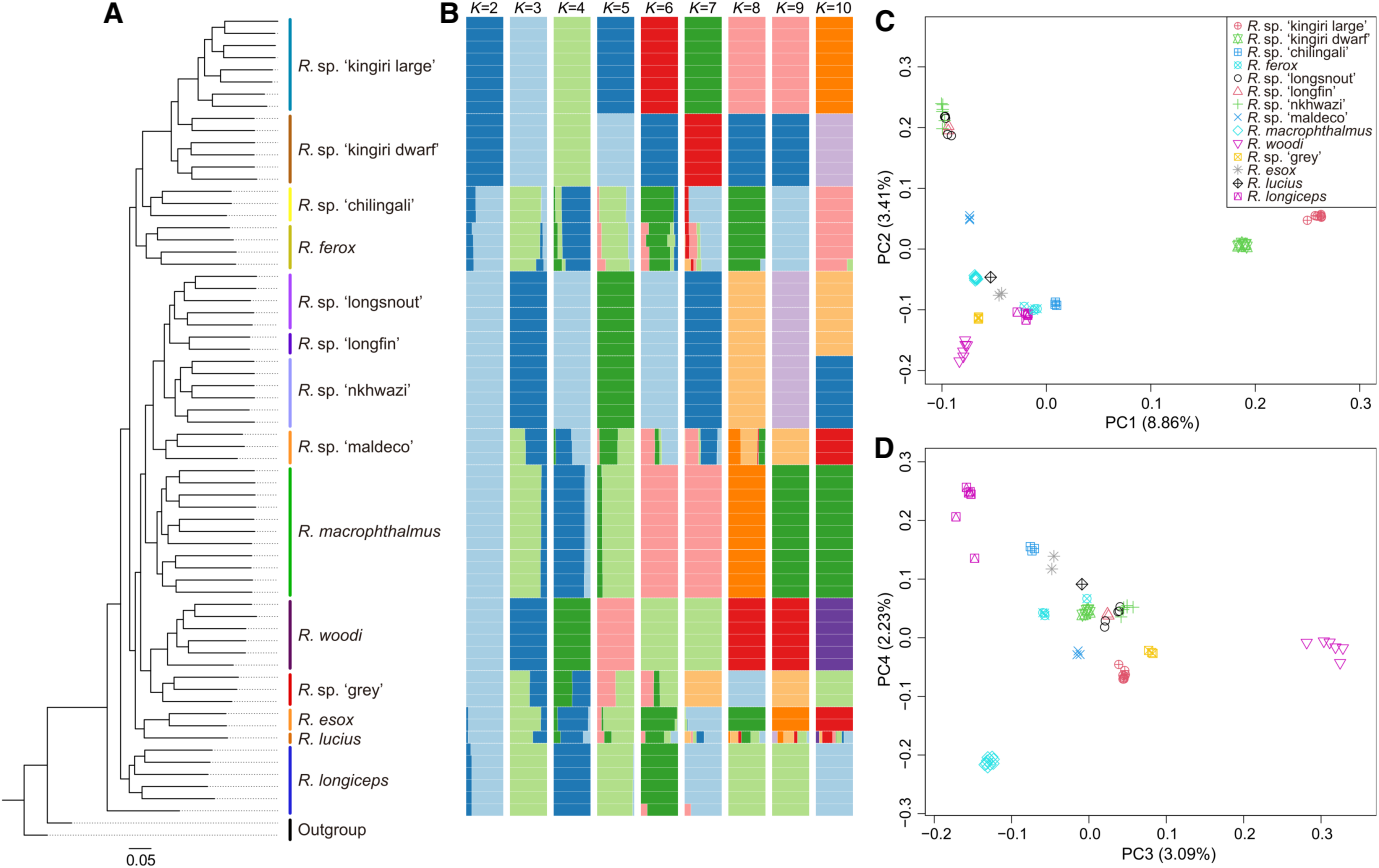
Phylogeny and population structure analysis of *Rhamphochromis* species. (*A*) Maximum likelihood phylogenetic tree reconstructed using whole-genome single-nucleotide polymorphism (SNP) data from all 66 *Rhamphochromis* individuals and two outgroup taxa. Bootstrap values are available in Supplemental Figure S1, and all nodes separating each species have a bootstrap of 100%. (*B*) Population structure of *Rhamphochromis* estimated using the ADMIXTURE program with admixture proportions of individuals from *K* = 2 to *K* = 10. Each color represents one population. Each individual is represented by a vertical bar, with the length of each colored segment indicating the proportion of genetic contribution from the respective ancestral population. (*C*,*D*) PCA plots of the first four components of *Rhamphochromis* individuals based on whole-genome SNP data.

To investigate how the *Rhamphochromis* radiation unfolded and the relationships between species with different body sizes, we first constructed a maximum likelihood (ML) phylogenetic tree based on the filtered high-quality SNPs found in the 68 individuals (Fig. 2A; Supplemental Fig. S1). Our phylogeny revealed that sampled Lake Malawi *Rhamphochromis* species formed distinct, reciprocally monophyletic groups with 100% bootstrap support, therefore resolving ambiguities present in a previous study based on mtDNA sequences (Genner et al. 2007). Species with similar body sizes did not cluster together, indicating that body size variation has evolved independently multiple times within the genus (Supplemental Fig. S1). Of particular interest are the two endemic species from Lake Kingiri, *Rhamphochromis* sp. “kingiri large” and *Rhamphochromis* sp. “kingiri dwarf,” that were resolved as sister species. Their closest relative is the small-bodied *Rhamphochromis* sp. “chilingali” from Lake Chilingali. These findings are consistent with the two endemic species in Lake Kingiri representing a case of sympatric speciation, distinguished by substantial phenotypic divergence, particularly in body size. As is typical for Malawi cichlids, the ML phylogenetic tree based on mitochondrial DNA (mtDNA) did not adequately capture the phylogenetic relationships resolved by the whole-nuclear-genome data (Supplemental Figs. S2, S3; Supplemental Table S4). Discrepancies likely reflect extensive incomplete lineage sorting (ILS) and introgression events during the evolutionary history of the radiation (Malinsky et al. 2018).

We investigated genome-wide population structure by inferring ancestry proportions for all *Rhamphochromis* species using ADMIXTURE (Supplemental Fig. S4; Alexander et al. 2009). This confirmed the presence of strong population structure among samples, with most species uniquely distinguished when *K* = 10, with the exception of two closely related species pairs (*Rhamphochromis* sp. “longfin” and *Rhamphochromis* sp. “longsnout”; *Rhamphochromis* sp. “chilingali” and *Rhamphochromis ferox*), one relatively distantly related pair (*Rhamphochromis* sp. “maldeco” and *Rhamphochromis esox*), and the one individual from *R. lucius* that had an unresolved placement (Fig. 2B; Supplemental Fig. S5). We further visualized population structure using principal component analysis (PCA) (Fig. 2C,D), which showed strong clustering by species across the first four axes. Notably, the first component (PC1) clearly separated the two species from Lake Kingiri from the species in Lake Chilingali and Lake Malawi, suggesting that the Lake Kingiri species share a more similar genetic background with one another than other *Rhamphochromis* species. This finding is consistent with the results from the ML phylogenetic analysis (Fig. 2A) and model-based analyses of population admixture (*K* = 2) (Fig. 2B).

To explore changes in effective population size (*Ne*) of the ancestral populations for each of the genome-sequenced *Rhamphochromis* species, we used a pairwise sequentially Markovian coalescent analysis (PSMC) (Supplemental Figs. S6, S7; Li and Durbin 2011). The analysis was based on a mutation rate of 3.5 × 10^−9^ mutations per site per year and a generation time of 3 years (Malinsky et al. 2018). Our results revealed substantial variation in effective population size across the different species, with *Rhamphochromis* sp. “kingiri large” and *Rhamphochromis* sp. “kingiri dwarf” from Lake Kingiri showing the smallest effective population sizes, less than 1 × 10^4^, consistent with their isolation in the small lake (600 m in diameter). Notably, nearly all Lake Malawi species exhibited a period of rapid population expansion that initiated within the past 100,000 years (Supplemental Figs. S6, S7).

### Genome-wide association study of the body size evolution

**Figure 3.**
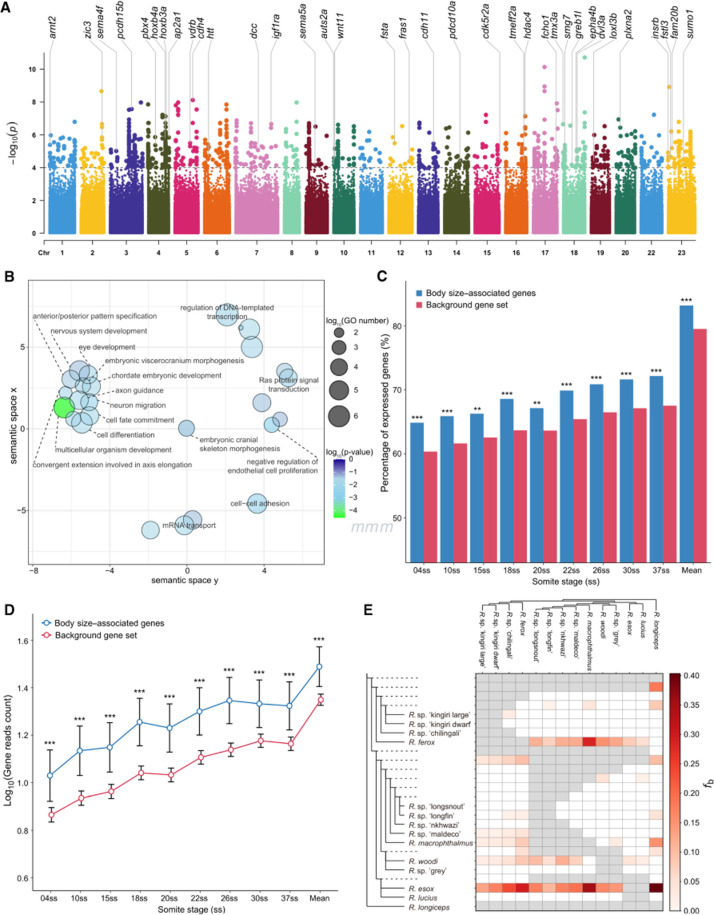
Association of skeletal and nervous system development with body size evolution. (*A*) GWA results for the body size for all 2,816,637 polymorphic SNPs within the *Rhamphochromis* radiation across 66 individuals. The *y*-axis corresponds to two-tailed −log_10_(*P*-values); the *x*-axis corresponds to genomic coordinates (fAstCal1.2). The horizontal dashed line reflects the threshold for calling genome-wide significant SNPs (*P* = 1 × 10^−4^). (*B*) Gene Ontology (GO) scatterplot constructed using REVIGO for all the GO terms and genes within the identified significant SNPs for body size. Bubble size indicates the relative frequency of the GO terms, and bubble color represents log_10_ *P*-values (smaller values indicate lower *P*-values). (*C*,*D*) Transcriptomic analysis of the genes significantly associated with body size evolution during early embryogenesis of *Rhamphochromis* sp. “chilingali,” showing higher proportion of expression (*C*) and higher expression level (*D*) for genes associated with body size evolution. (*E*) Heatmap showing the estimated admixture fractions between pairs of species represented on the *x*- and *y*-axes. Species are arranged along the axes according to their placement in the species tree. The matrix values (*f*_b_) were calculated using Dsuite's *f*-branch statistic, which quantifies excess allele sharing between the branch specified on the expanded tree along the *y*-axis (relative to its sister branch) and the species identified on the *x*-axis. These values provide insights into the hybrid origin of shared alleles. Gray squares indicate comparisons that cannot be made.

To examine the genetic basis of body size evolution, we conducted a genome-wide association (GWA) mapping analysis on body size (standard length) across all 66 *Rhamphochromis* individuals, accounting for population structure by incorporating PCA covariates using PLINK v.1.9072 (Purcell et al. 2007). This analysis identified 1347 SNPs (*P* < 0.0001) significantly associated with body size, linked to 570 genes (Fig. 3A; Supplemental Fig. S8; Supplemental Table S5). Notably, several of these genes, including *hoxb4a*, *hoxb3a*, *wnt11*, and *igf1ra*, are involved in developmental processes critical for embryonic growth and body size regulation. These genes are known for their roles in pathways controlling cell differentiation, tissue patterning, and growth, suggesting that body size evolution in *Rhamphochromis* is tightly linked to genetic networks governing early developmental stages and morphogenesis. To validate the robustness of our results using independent methods and data sets, we conducted GWA analyses using linear mixed models (LMM) implemented using FaSt-LMM in GWAStic (Lück et al. 2024), using an automatically generated kinship matrix to account for population structure. One LMM analysis used standard length as the response variable, whereas a second LMM analysis using log_10_-transformed standard length as the response variable. The two independent analyses identified 2005 and 4481 SNPs (*P* < 0.0001), respectively, significantly associated with body size (Supplemental Tables S6, S7). The overlap between these results and those obtained from PLINK was substantially higher than expected by chance (1347 standard-length PLINK SNPs, 2005 SNPs standard-length LMM SNPs, overlap 766 SNPs, hypergeometric test, *P* < 0.001; 1347 standard-length PLINK SNPs, 4481 SNPs log_10_ standard-length LMM SNPs, overlap 340 SNPs, hypergeometric test, *P* < 0.001).

To investigate whether the GWA-identified genes were enriched for specific Gene Ontology (GO) terms, we identified one-to-one orthologs between *Astatotilapia calliptera* and zebrafish, before conducting GO enrichment analyses using DAVID (Fig. 3B; Supplemental Table S8). This analysis revealed that these 570 body size–associated genes are enriched in GO terms related to embryonic development and skeletal morphogenesis. Notable examples include “anterior/posterior pattern specification” (GO:0009952), “embryonic viscerocranium morphogenesis” (GO:0048703), “multicellular organism development” (GO:0007275), and “embryonic cranial skeleton morphogenesis” (GO:0048701). These findings underscore the pivotal roles of these genes in key developmental processes, highlighting their potential involvement in the regulation of body size evolution. Moreover, the enrichment of these specific GO terms suggests that body size divergence in *Rhamphochromis* may be driven by modifications to conserved developmental pathways.

To further investigate the functional roles of the GWA-identified genes, we examined their expression patterns during the embryonic development of *Rhamphochromis* sp. “chilingali” using published expression data from whole embryos (Marconi et al. 2024). Notably, we found that body size–associated genes (GWA-identified genes) were significantly more likely to be expressed during development in *Rhamphochromis* sp. “chilingali” than other genes not associated with body size, across embryonic developmental stages (Fig. 3C; Supplemental Table S9). Additionally, the expression levels of GWA-identified genes were higher than those of other genes across embryonic developmental stages (Fig. 3D). These findings suggest that the genes associated with body size in *Rhamphochromis* species may take active roles during early development, which is key period for establishing body plan proportions in vertebrates.

### Hybridization during the evolution of the genus *Rhamphochromis*

Discordance between phylogenetic trees constructed from nuclear and mitochondrial sequence data has been reported in many taxa (Gompert et al. 2008; Yang et al. 2021; Du et al. 2024), which may reflect a history of interspecific gene flow, such as hybridization, or result from processes like ILS (Rivas-González et al. 2023; Steenwyk et al. 2023). With complete genome resources now available, it is feasible to distinguish between the effects of hybridization and ILS by applying *f*_4_-ratio statistics using the “*f*-branch” method (Malinsky et al. 2021). In *Rhamphochromis*, *f*-branch statistics revealed several cases of interspecific gene flow, highlighted by the orange-red shading in the heatmaps (Fig. 3E). Most notably, the nuclear genome of *R. esox* contained high admixture proportions with several groups, including *Rhamphochromis longiceps*, an ancestor of the lineage containing *R. ferox*, and an ancestor of the lineage containing *Rhamphochromis macrophthalmus* (Fig. 3E). This pattern was apparent in both sequenced individuals of *R. esox* (Supplemental Fig. S9), consistent with a hybrid origin of *R. esox* from multiple hybridization events. Other apparent hybridization events were apparent between *R. fero*x and *R. macrophthalmus* and between *R. longiceps* and *R. macrophthalmus* (Fig. 3E).

### Sympatric speciation of two species of *Rhamphochromis* in Lake Kingiri

**Figure 4.**
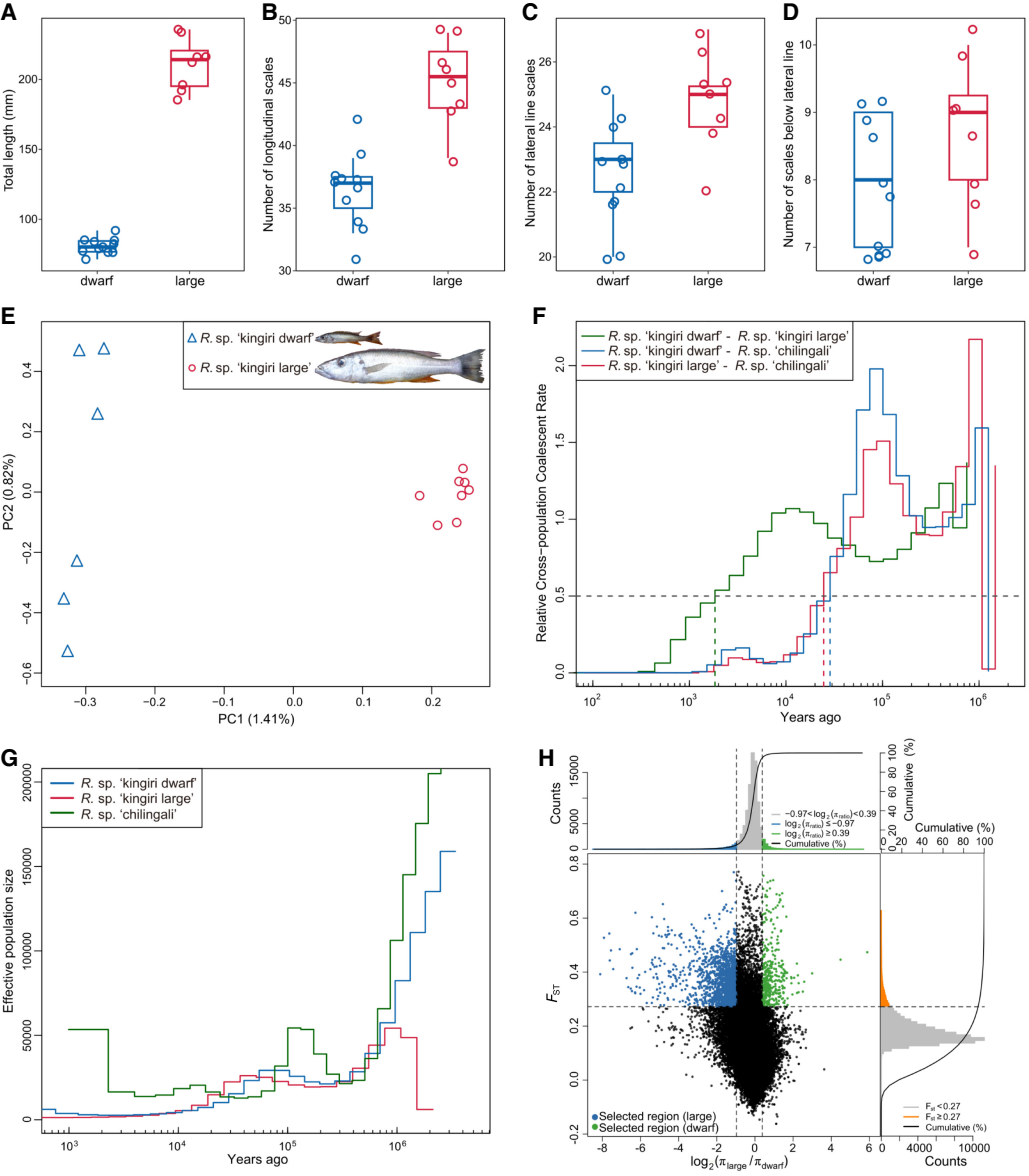
Morphological and genomic divergence between sympatric dwarf and large *Rhamphochromis* species in Lake Kingiri. (*A*–*D*) Relative to *Rhamphochromis* sp. “kingiri large,” *Rhamphochromis* sp. “kingiri dwarf” has smaller total length (*A*), fewer longitudinal scales (*B*), fewer lateral line scales (*C*), and fewer scales below the lateral line (*D*). (*E*) Principal component analysis for all Lake Kingiri *Rhamphochromis* using whole-genome SNP data. (*F*) MSMC2 cross-coalescence between *Rhamphochromis* sp. “kingiri dwarf” and *Rhamphochromis* sp. “kingiri large” individuals (green line), and between Lake Kingiri *Rhamphochromis* species and *Rhamphochromis* sp. “chilingali” (blue and red lines). Dotted lines indicate the split time for each population pair (relative cross-coalescence rates = 0.5). (*G*) Demographic history and effective population size of the two *Rhamphochromis* species in Lake Kingiri (blue and red lines) and the species from Lake Chilingali (green line) using MSMC2. (*H*) Distribution of log_2_ ratio of nucleotide diversity (π; π_large_/π_dwarf_) and *F*_ST_ of 50 kb windows with 10 kb steps. Colored data points indicate selected windows for *Rhamphochromis* sp. “kingiri large” (in blue) and *R*. “kingiri dwarf” (in green). Vertical lines reflect the 5% left and right tails of the log_2_(π_large_/π_dwarf_) distribution (thresholds –0.97 and 0.39, respectively). The horizontal dashed line reflects the top 5% of windows in the *F*_ST_ distribution (threshold *F*_ST_ = 0.27).

Lake Kingiri is an isolated crater lake situated 21.3 km northwest from the northernmost point of Lake Malawi (∼600 m diameter, 34 m deep) (Supplemental Fig. S10; Turner et al. 2019). The two *Rhamphochromis* species present differ in key phenotypic traits (Supplemental Fig. S11; Supplemental Table S2), with *Rhamphochromis* sp. “kingiri dwarf” being significantly smaller than *Rhamphochromis* sp. “kingiri large” (Wilcoxon signed-rank test, *P* = 6.17 × 10^−8^) (Fig. 4A). Additionally, *Rhamphochromis* sp. “kingiri dwarf” exhibits significantly fewer longitudinal scales, and significantly fewer upper lateral line scales, compared to *Rhamphochromis* sp. “kingiri large” (Wilcoxon signed-rank test, *P* = 4.83 × 10^−5^, *P* = 0.0068, respectively) (Fig. 4B–D).

Analysis of sequences are consistent with the two species being reproductively isolated sympatric species pair (Fig. 4E; for admixture plots on *K* = 5 or higher, see Fig. 2B), with the genetic diversity of *Rhamphochromis* sp. “kingiri large” being slightly lower than that of *Rhamphochromis* sp. “kingiri dwarf” (Supplemental Fig. S12). Cross-coalescence rate analysis using the multiple sequentially Markovian coalescent (MSMC2) indicated that the divergence between *Rhamphochromis* sp. “kingiri dwarf” and *Rhamphochromis* sp. “kingiri large” occurred <2000 years ago, whereas the divergence between the ancestor of the *Rhamphochromis* from Lake Kingiri and the ancestor of the *Rhamphochromis* from Lake Chilingali took place ∼25,000 years ago (Fig. 4F). Effective population size estimates from MSMC2 revealed small population sizes for *Rhamphochromis* sp. “chilingali,” *Rhamphochromis* sp. “kingiri dwarf,” and *Rhamphochromis* sp. “kingiri large,” with the smallest effective population size in the Lake Kingiri species (Fig. 4G), generally consistent with estimations using PSMC (Supplemental Figs. S6, S7).

### Genomic islands of speciation

Genome-wide divergence for *Rhamphochromis* sp. “kingiri dwarf” and *Rhamphochromis* sp. “kingiri large” was *F*_ST_ = 0.067, with almost half of variable sites having zero *F*_ST_ (48.6%) (Supplemental Table S10). The maximum *F*_ST_ for a single SNP site is 6.2 SD above the mean *F*_ST_, with 10,048 SNP sites having *F*_ST_ above 4 SD of mean *F*_ST_ (Supplemental Figs. S13, S14). Relative divergence (*ZF*_ST_) values identified 438 nonoverlapping genomic islands, merged into in 88 contiguous islands, between *Rhamphochromis* sp. “kingiri dwarf” and *Rhamphochromis* sp. “kingiri large.” Of these contiguous islands, 21 were ≥100 kb, and these 21 accounting for 48.84% of total genomic island length (Supplemental Table S11; Supplemental Fig. S14). In comparison to the background, genomic islands exhibited significantly higher levels of absolute divergence (*D*_xy_; Mann–Whitney *U* test; *P* < 2.2 × 10^−16^) (Supplemental Figs. S14, S15). This aligns with speciation-with-gene-flow models, which predict that loci involved in speciation should display both high relative divergence (*F*_ST_) and high absolute sequence divergence (*D*_xy_) (Noor and Bennett 2009; Cruickshank and Hahn 2014; Malinsky et al. 2015). Similar patterns were observed regardless of whether the *ZF*_ST_ cutoff was set at 3.5 or three (Supplemental Table S11; Supplemental Fig. S16). Meanwhile, we found significantly reduced π for both Kingiri taxa in these islands compared with the rest of the genome (Mann–Whitney *U* test; *P* < 1.5 × 10^−5^) (Supplemental Fig. S17).

### Selection signals in the lake Kingiri sympatric species pair

We combined *F*_ST_ and π analyses between the two sympatric taxa to investigate selection underlying the phenotypic divergence (Fig. 4H). In total, we identified 1081 genes from 1648 putatively selected regions in *Rhamphochromis* sp. “kingiri large” (Supplemental Table S12), considerably higher than the 242 genes from 401 putatively selected regions in *Rhamphochromis* sp. “kingiri dwarf” (Supplemental Table S13). This indicates that *Rhamphochromis* sp. “kingiri large” has experienced stronger divergent selection compared with *Rhamphochromis* sp. “kingiri dwarf,” an interpretation consistent with the strongly reduced π in *Rhamphochromis* sp. “kingiri large” (Supplemental Fig. S12). We also found that the overlap between these selected genes within the Lake Kingiri populations and the GWA-identified body size–associated genes was significantly greater than expected by chance. This was the case for both populations (1081 *Rhamphochromis* sp. “kingiri large” selected genes 570 GWAS selected genes, overlap 47 genes, hypergeometric test, *P* = 2.76 × 10^−6^; 242 *Rhamphochromis* sp. “kingiri dwarf” selected genes, 570 GWAS selected genes, overlap 16 genes, hypergeometric test, *P* = 6.31 × 10^−5^).

**Figure 5.**
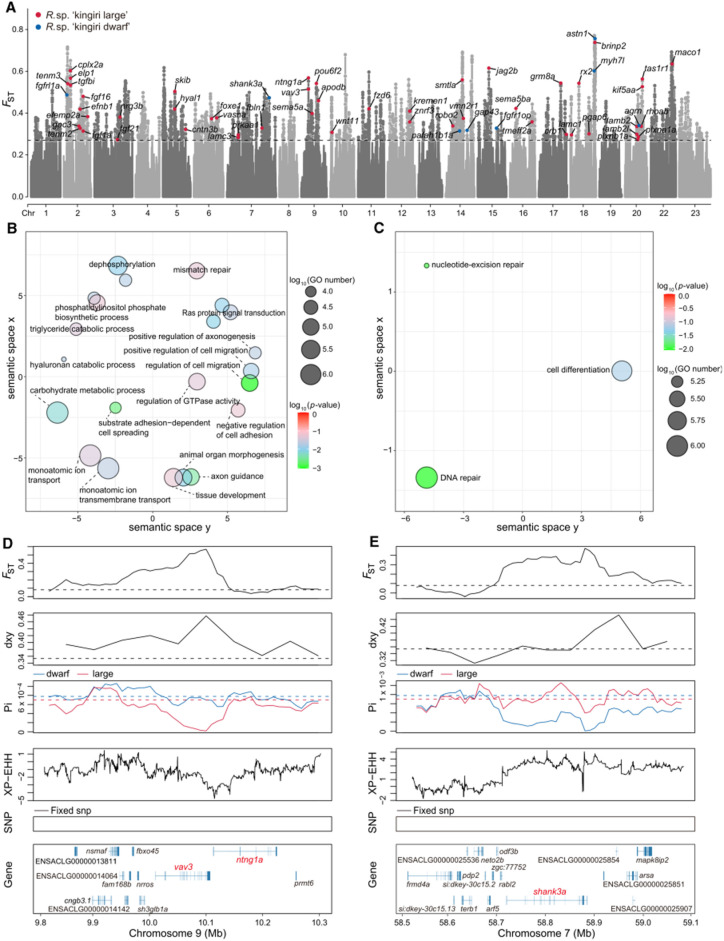
Selection analyses of the sympatric large and dwarf species of *Rhamphochromis* in Lake Kingiri. (*A*) Manhattan plot of the pairwise fixation index (*F*_ST_) between *Rhamphochromis* sp. “kingiri large” and *Rhamphochromis* sp. “kingiri dwarf,” with selected genes in *Rhamphochromis* sp. “kingiri large” in red and selected genes in *Rhamphochromis* sp. “kingiri dwarf” in blue. The dashed gray line represents the up 5% *F*_ST_ threshold of 0.27. (*B*,*C*) GO scatterplots constructed using REVIGO for all the GO terms and genes identified as selected genes in *Rhamphochromis* sp. “kingiri large” (*B*) and genes identified as selected genes in *Rhamphochromis* sp. “kingiri dwarf” (*C*). Bubble size indicates the relative frequency of the GO terms and bubble color represents log_10_ *P*-values (smaller values indicate lower *P*-values). (*D*,*E*) Selective sweep on Chromosome 9 (9.8 to 10.3 Mb; *D*) and selective sweep on Chromosome 7 (58.5 to 59.1 Mb; *E*). The putative sweep region is validated by *F*_ST_, *D*_xy_, Pi, and XP-EHH test. Horizontal dashed lines represent the genome-wide means for the corresponding parameters. Gene annotations in the sweep region and SNPs nearly fixed for derived alleles are indicated at the *bottom*.

Many genes with functional roles in tissue development and organ morphogenesis were identified as selected genes in *Rhamphochromis* sp. “kingiri large,” whereas only a few such genes were estimated to be under recent selection in *Rhamphochromis* sp. “kingiri dwarf” (Fig. 5A). For example, the genes, *fgfrl1a*, *fgf21*, *wnt11*, and *fgfr1op*, which are all involved in organ development, were shown as under recent selection in *Rhamphochromis* sp. “kingiri large.” Consistent with this, GO analysis of these 1081 selected genes in *Rhamphochromis* sp. “kingiri large” indicated that they were significantly enriched for regulation of cell migration, animal organ morphogenesis, axon guidance, and tissue development (Fig. 5B; Supplemental Table S14). However, only three GO terms were significantly enriched for the 242 selected genes in *Rhamphochromis* sp. “kingiri dwarf,” including nucleotide-excision repair, cell differentiation, and DNA repair (Fig. 5C; Supplemental Table S15). Among the selected genes, we observed that both *F*_ST_ and *D*_xy_ are particularly high in the *VAV3* genic region. Here, six missense single-nucleotide polymorphisms (SNPs) were also identified, exhibiting *F*_ST_-values greater than 0.5, and XP-EHH scores below –4.8 (Fig. 5D; Supplemental Fig. S18). The genetic diversity estimator π is significantly lower in *Rhamphochromis* sp. “kingiri large” for this gene, indicating it has recently been under selection in this species. Another gene with a strong signal of selection is *shank3a*. *F*_ST_, *D*_xy_, and XP-EHH are all markedly elevated in the *shank3a* region, suggesting strong positive selection at this locus. Additionally, a significantly reduced nucleotide diversity (π) is observed in *Rhamphochromis* sp. “kingiri dwarf,” indicates that the *shank3a* developmental gene is under strong selection in this species.

## Discussion

### Genetic structure in *Rhamphochromis*

To investigate how the radiation of this genus of pelagic predators unfolded, we generated the first comprehensive phylogenetic reconstruction using whole-genome data. Our analyses of nuclear genomes span 14 of the 15 species that comprise the radiation, the exception being the relatively rare *Rhamphochromis* sp. “longiceps yellow belly” (Genner et al. 2007; Konings 2016), from which we were unable to obtain sufficient nuclear genome data for inclusion. Our results clarify the species richness of the genus and are supported by combination of morphological characteristics, as well male breeding colors (Genner et al. 2007), which is meaningful given that *Rhamphochromis* species as a whole are considered one of the most challenging groups within the Lake Malawi cichlid radiation to reliably identify to species. In total, nine of the 15 species in the radiation remain undescribed, including the largest cichlid species in the Lake Malawi radiation, here referred to as *Rhamphochromis* sp. “nkhwazi,” and the one of the smallest species in the entire radiation, *Rhamphochromis* sp. “kingiri dwarf.” Our clear evidence of the genetic distinction of these nine species will inform taxonomic descriptions of the group, beneficial as it would enable IUCN conservation assessments. This is important as all *Rhamphochromis* in Lake Malawi are targeted species in commercial fisheries. Meanwhile, the satellite lake species are also highly threatened owing to their restricted ranges, including *Rhamphochromis* sp. “chilingali,” which is now believed to be extinct in the wild following the desiccation of Lake Chilingali in 2012 (Turner et al. 2023), and *Rhamphochromis* sp. “kingiri large,” which was the target of an artisanal gillnet fishery when last sampled in 2011 (Turner et al. 2019).

### Genetic basis of body size diversity in *Rhamphochromis*

Our phylogenetic analyses based on nuclear genomes reveal the evolution of body size diversity in *Rhamphochromis*. Specifically, we were able to reconstruct repeated shifts in body size, as indicated by the small-bodied species including *R. longiceps*, *R. macrophthalmus*, and *Rhamphochromis* sp. “chilingali” being dispersed across the phylogeny. This dynamic body size evolution is a broader characteristic of fish radiations in cichlids and is strongly associated with diet, including in *Rhamphochromis*. The smallest *Rhamphochromis*, including *Rhamphochromis* sp. “kingiri dwarf” and *Rhamphochromis* sp. “chilingali” feed upon zooplankton and very small fish. Meanwhile, medium-size species such as *R. longiceps* forage on zooplankton and shoaling cyprinids (Allison et al. 1996). The largest of all *Rhamphochromis* are specialist cichlid predators, feeding primarily on deep water species such as *Diplotaxodon* (Allison et al. 1996). The strong link between body size and diet in *Rhamphochromis* is therefore reflective of other fish radiations with notable body size diversity, such as Arctic charr (Malmquist et al. 1992) and coregonid whitefishes (Østbye et al. 2006).

The extensive shared genetic variation among species within African cichlid radiations makes them useful for the identification of genetic variants associated with phenotypic traits (Sommer-Trembo et al. 2024). Our GWA analysis identified numerous genes that were strongly associated with body size in *Rhamphochromis*, consistent with a highly polygenic basis to body size divergence in cichlids, as observed in other vertebrate groups, including mammals (Bouwman et al. 2018) and birds (Luo et al. 2025). It is plausible that many of the genes we identified are only indirectly associated with body size, for example, being associated with covarying environmental variables including water depth or diet. Nevertheless, because our analyses identified suites of genes associated with morphogenesis and embryonic development and because those genes tended to exhibit elevated levels of expression during larval development, then we have confidence that our analysis has identified relevant candidate genes underpinning body size evolution. Importantly, multiple candidate genes identified in this study have previously been identified in body size regulation in other studies of vertebrates. For example, *hoxb3a* is expressed in skeletal development of three-spined stickleback *Gasterosteus aculeatus* (Ahn and Gibson 1999) and has been invoked as a potential contributor to the development of a giant phenotype of the Siamese fighting fish *Betta splendens* (Wang et al. 2022). Meanwhile *igf1ra*, an insulin-like growth factor receptor orthologous to *igf1r*, is a key regulator of morphological development in zebrafish (Schlueter et al. 2007) and has been found to be under selection in the giant ocean sunfish *Mola mola* (Pan et al. 2016), as well as being linked to growth variants in the striped catfish *Pangasianodon hypophthalmus* (Tran et al. 2021). Moreover, the insulin-like growth factor 1, which activates *igf1r*, is known to be a primary regular of growth hormone, as well as a mediator of body size in multiple vertebrate groups (Sutter et al. 2007; Kappeler et al. 2008). Collectively, this GWA analysis had indicated considerable potential for further investigation of the body size evolution using *Rhamphochromis*, as well as potentially other haplochromine cichlid radiations, that could be validated using QTL-mapping approaches and genetic manipulation methods (Santos et al. 2023).

### Genomic insights into the evolution of the Lake Malawi *Rhamphochromis* radiation

The genus *Rhamphochromis* is part of a distinct pelagic-dwelling subtribe within the Lake Malawi radiation. Most of the species-specific signals of population expansion, as inferred by PSMC analyses, began ∼400,000 years ago, potentially indicative of the start of diversification within the subtribe. This time line broadly fits with the upper limits of the estimated start of the radiation dating to 860,000–990,000 years ago (Malinsky et al. 2018). Notably most of the main lake populations show expansions over the past 100,000 years, coinciding with a major rise in the level of Lake Malawi to present levels (Lyons et al. 2015), potentially reflecting pelagic habitat expansion and novel ecological opportunity after a severe lowstand.

Our study has revealed several instances of admixture within *Rhamphochromis*, for example, between *R. esox* and *R. longiceps* (also seen in Malinsky et al. 2018) and between *R. fero*x and *R. macrophthalmus*. These events have plausibly led to sharing of adaptive alleles, potentially promoting radiation in the lineage, as has been proposed in other examples of ancestral gene flow in Lake Victoria (Meier et al. 2023), as well as Lake Malawi (Malinsky et al. 2018). Although cases of admixture between major lineages of cichlid fishes in Lake Malawi have been revealed through whole-genome sequencing (Malinsky et al. 2018; Blumer et al. 2025; Kumar et al. 2025), there is currently no evidence of horizontal gene flow between *Rhamphochromis* and other Lake Malawi cichlid lineages (Malinsky et al. 2018), perhaps reflective of the distinctive pelagic predator ecology of *Rhamphochromis* and their ability to breed in open water, which is uncommon in Malawi cichlids.

### Sympatric speciation in Lake Kingiri

The two sympatric *Rhamphochromis* species in Lake Kingiri, *Rhamphochromis* sp. “kingiri large” and *Rhamphochromis* sp. “kingiri dwarf,” were found to be morphologically and genetically divergent sister species. Both species had very small effective population sizes, including evidence of historic reductions in the population size of the ancestral species which may be expected following containment in the crater lake. Notably, we estimated these have diverged in the past 2000 years from common ancestry. The precise age of Lake Kingiri is not known, but it is one of several maar crater lakes in the region, and the geographically proximate Lake Masoko has been dated to 50,000 years old based on carbon dating of drill core material (Williamson et al. 1999), and it is plausible Lake Kingiri was formed during the same period of volcanic activity.

The mechanism of colonization of Lake Kingiri is unclear, but it was likely colonized directly from the proximate River Mguwesi. Lake Kingiri currently has a linear distance of 16 km from the current shoreline of Lake Malawi and, with a surface elevation of 523 m, is currently ∼50 m above the current Lake Malawi level of ∼473 m. Lacustrine sediments (raised beaches) provide evidence of Lake Malawi stands ∼40 m higher than present-day levels in the upper Pleistocene (Pike 1958; Clark et al. 1966). Hence, Lake Malawi was historically in very close proximity to Lake Kingiri, which may have facilitated colonization of the crater lake by the ancestor of the *Rhamphochromis* species pair during or after the separation from *Rhamphochromis* sp. “chilingali” ∼25,000 years ago.

Although it is possible that the Lake Kingiri was colonized on multiple occasions, each time leading to a speciation event from a common Lake Malawi ancestor, most parsimoniously, the divergence was a result from one common ancestor, as this would require only one extinction event of a main lake ancestor since separation from *Rhamphochromis* sp. “chilingali.” Demographic modeling may help to clarify the most likely evolutionary scenario (Kautt et al. 2016). Importantly, neither of the Lake Kingiri species shows any clear signs of admixture with Lake Malawi or Lake Chilingali species. The slightly closer position of *Rhamphochromis* sp. “kingiri dwarf” compared with *Rhamphochromis* sp. “kingiri large” to the most genetically similar species *Rhamphochromis* sp. “chilingali” in the PCA plot is consistent with differential influence of genetic drift or selection on the populations after divergence from *Rhamphochromis* sp. “chilingali.” Notably *R*. “kingiri dwarf” has a higher effective population size, and from a phylogenetic perspective, the nested position of *Rhamphochromis* sp. “kingiri large” within *Rhamphochromis* “kingiri dwarf” is indicative that *Rhamphochromis* sp. “kingiri large” may be the derived species.

The Lake Kingiri speciation event is remarkable for the divergence in body size when contrasted with other cichlid fish crater lake speciation events. In nearby Lake Masoko, the two ecotypes of *A. calliptera* have diverged most patently in trophic morphology and male breeding colors rather than size (Malinsky et al. 2015). Similarly, species from the crater lake cichlid radiations in Cameroon and Nicaragua tend to vary primarily in body shape, as well as tooth and jaw morphology, with conspicuous contrasts in adult body size less common (Trewavas et al. 1972; Stauffer et al. 2008; Dunz and Schliewen 2010). We speculate that the speciation event in Lake Kingiri has followed similar selection pressures to the size-based diversification of predatory ecotypes seen in coregonid whitefish (Lu and Bernatchez 1999) and Arctic charr (Doenz et al. 2019). In those systems, the availability of distinct prey fields, coupled with strong competition, is proposed to lead to selection favoring multiple distinct size-differentiated ecotypes (Salisbury and Ruzzante 2022). Speciation may further be promoted by targeted selection, including from predators (Öhlund et al. 2020), leading ecotypes to occupy different breeding habitats or have asynchronous breeding schedules. In Lake Kingiri, adult *Rhamphochromis* sp. “kingiri dwarf” are commonplace in shallow littoral habitat, whereas adult *Rhamphochromis* sp. “kingiri large” have only been captured offshore deeper water, suggestive of different patterns of habitat occupancy of breeding fish. We also speculate that this reproductive isolation may be reinforced if larger individuals are perceived as potential predators by breeding *Rhamphochromis* sp. “kingiri dwarf” and if smaller individuals are perceived as potential prey by breeding *Rhamphochromis* sp. “kingiri large.”

We observed islands of genomic differentiation between *Rhamphochromis* sp. “kingiri dwarf” and *Rhamphochromis* sp. “kingiri large,” in a pattern consistent with a relative recent divergence driven by selection (Malinsky et al. 2015). Our subsequent analyses showed stronger selection operating on *Rhamphochromis* sp. “kingiri large” relative to *Rhamphochromis* sp. “kingiri dwarf.” This is compatible with the concept that *Rhamphochromis* sp. “kingiri large” has evolved from a small-bodied ancestor, which perhaps was phenotypically similar to the closely related dwarf species *Rhamphochromis* sp. “chilingali.” One interpretation of the evidence is that an ancestral small-bodied species once had a much wider distribution around Lake Malawi and was specialized for shallow marginal habitats but was ultimately extirpated from the main lake body. However, this ancestral lineage persisted in satellite Lake Chilingali and Lake Kingiri, finding ecological opportunity to speciate only in the deeper Lake Kingiri.

Our analyses revealed large numbers of genes experiencing selection in Lake Kingiri. Given overlap with the genes identified in GWA analyses, many are likely to be associated with divergence in body size, whereas others may be associated with other characteristics linked to their speciation. Genes under selection were linked to skeletal, organ, and neurological development. A number of genes with evidence of selection were associated with fibroblast growth factors, including *fgfrl1a*, a gene linked to gill cartilage development in zebrafish (Hall et al. 2006), and *fgf2*, a gene known to determine body size in Asian seabass *Lates calcarifer* (Wang et al. 2014). A further gene highlighted by the analysis was *Wnt11*, a regulator anterior-posterior axis elongation in mammals (Andre et al. 2015). We also found strong evidence for *VAV3* and *shank3a* being under positive selection in *Rhamphochromis* sp. “kingiri large” and *Rhamphochromis* sp. “kingiri dwarf,” respectively. *VAV3* encodes a guanine nucleotide exchange factor (GEF) for Rho family GTPases, which regulate key cellular processes such as cell growth, transformation, and development. In neurons, it plays a crucial role in axon elongation and dendrite formation (Sauzeau et al. 2010). Meanwhile, *shank3a* has a crucial role in brain development and function, particularly in synapse formation and maturation. Mutations in this gene have been associated with autism spectrum disorder (ASD) and Phelan–McDermid syndrome (Liu et al. 2022). Collectively, these analyses have provided multiple candidate genes suitable for further research into links between functional genetic variation and divergent phenotypic development.

Although limited in the number of sequences studied for each species, the use of only one reference genome for mapping, and our focus on body size, our study provides unprecedented insight into the evolution of a radiation of pelagic cichlid fishes. In particular, our study has highlighted how rapid speciation in the lineage associates with body size divergence. To date, the genetic basis of adaptive evolution in body size of cichlid fishes has been largely overlooked, yet this appears to be a key axis of ecological diversification across radiations in Lake Malawi, Lake Victoria, and Lake Tanganyika, as well as teleost fishes more broadly. There remains a considerable amount to learn about the ecology and adaptive radiation of pelagic cichlids, including their biogeography, habitat preferences, breeding behavior, and the genetic basis of other phenotypic traits, both in Lakes Malawi and Kingiri. Nonetheless, our results provide fundamental insight into their diversity, which will support future research into this ecologically, economically, and culturally important group of Lake Malawi cichlids.

## Methods

### Field sampling

*Rhamphochromis* samples from the Lake Malawi catchment area, including Lake Kingiri, Lake Chilingali, and Lake Malawi, were collected during multiple expeditions between 2004 and 2011. Fish were captured using fixed gill nets and scuba or obtained from local fishermen. Sampling was focused on individuals that were identifiable to species, based on anatomical and color traits. Upon retrieval, the fish were euthanized with an overdose of anesthetic (MS-222). For genetic analysis, fin clips were collected from each fish and preserved in ethanol. Sequence data were obtained at the level of the individual fish. For morphological measurements, whole specimens were fixed in ethanol or ∼4% formalin.

### Morphological analysis

For *Rhamphochromis* samples, standard length was measured, and the number of dorsal spines, dorsal rays, and anal rays were counted (Supplemental Table S1). For *Rhamphochromis* sp. “kingiri dwarf” and *Rhamphochromis* sp. “kingiri large,” additional meristic counts and length measurements were taken for 19 specimens: 11 *Rhamphochromis* sp. “kingiri dwarf” and eight *Rhamphochromis* sp. “kingiri large,” following the method of Snoeks (2004). These were total length, standard length, number of lateral line scales (longitudinal scales), scales above lateral line (upper transversal line scales), scales below lateral line (lower transversal line scales), lower jaw total outer tooth count, dorsal spine count, dorsal soft ray count, and anal spine count (Supplemental Table S2; following the method of Snoeks 2004). Length measurements were taken using digital calipers.

### DNA extraction and sequencing

Standard Qiagen DNeasy blood and tissue protocol was used to extract DNA from fin clips preserved in ethanol between 2004 and 2011. To ensure high-molecular-weight DNA was present in the final extraction products, samples were analyzed using Qubit, NanoDrop, and finally gel electrophoresis. High-quality samples were then chosen for whole-genome sequencing, ensuring an adequate scope across the *Rhamphochromis* genus. Final extracted products were then sequenced by Novogene using their standard whole-genome library prep and protocol. Sequencing was done using Illumina technology (150 bp paired-end) to a mean coverage of 10.3× (Supplemental Table S3). As the coverage of the sample of *Rhamphochromis* sp. “longiceps yellow belly” was only about 0.7× coverage, it was removed for whole-genome phylogenetic analysis and only used for mitochondrial genome analysis.

### Read mapping and variant calling

A total of 48 *Rhamphochromis* individuals were sequenced in this study, with an additional 21 obtained from NCBI: 19 *Rhamphochromis* and two outgroups (Supplemental Table S3). Low-quality reads were first filtered using fastp with default parameters (Chen et al. 2018). Then, the high-quality reads were aligned to the reference genome of *A. calliptera* (fAstCal1.2) from Ensembl (Release 111) using Burrows–Wheeler aligner (BWA) v0.7.16a-r1181 (Li and Durbin 2009), with the parameter “bwa mem -t 10 R.” This reference species is another member of the Lake Malawi radiation and should be approximately genetically equidistant to all representatives of the focal genus *Rhamphochromis* limiting reference bias. SAMtools v1.9 (Li and Durbin 2011) was used to sort and merge files, as well as to convert mapping outputs to binary alignment map (BAM) format. Subsequent steps, including local realignment and duplicate read marking, were carried out using Picard tools (version 2.1.1; http://broadinstitute.github.io/picard/). Variant calling for each sample was conducted with the Genome Analysis Toolkit (GATK) v4.1.1.0 (McKenna et al. 2010). Specifically, GATK's HaplotypeCaller was employed to identify variants for each accession. Additionally, a joint genotyping process was performed on genome variant call format (gVCF) files to produce a unified set of variations. To minimize the occurrence of false-positive SNPs, variants were filtered using GATK v4.1.1.0 (McKenna et al. 2010) and VCFtools v1.13 (Danecek et al. 2011) with the cutoff of “QUAL < 30.0 || QD < 2.0 || FS > 60.0 || MQ < 40.0 || SOR > 4.0” to discard site types probably caused by mapping bias and following cutoffs: minimum depth >20, minor allele frequency (MAF) >0.05, missing rate <0.5, and only keep biallelic sites. Finally, a total of 2,816,637 sites that passed all these filtering criteria were used in further phylogenetic analyses.

### Phylogenetics, admixture, and PCA analyses

A phylogenetic approach was first used to investigate the relationships between each of the *Rhamphochromis* species. The filtered VCF variant file was converted to a PHYLIP file using vcf2phylip v.277 (https://zenodo.org/records/2540861), and the ML tree was constructed using iqtree2 (Minh et al. 2020) with default parameters. FigTree v.1.4.3 (http://tree.bio.ed.ac.uk/software/fgtree/) was used to display the ML tree. Admixture analysis was assessed using the default setting in the ADMIXTURE v.1.3.0 (Alexander et al. 2009) with the number of assumed genetic clusters *K* ranging from two to 12, specifying 20 cross-validations (-cv = 20). PCA for all 66 individuals was performed by PLINK v.1.9072 (Purcell et al. 2007) with the parameter “‐‐pca.”

### Phylogenetic relationships of mitochondrial genomes

The mitochondrial genome of each individual was de novo assembled using MitoFinder (Allio et al. 2020), with *A. calliptera* (obtained from the NCBI Nucleotide database [https://www.ncbi.nlm.nih.gov/nucleotide/] under accession NC_018560) set as a starting reference. For information on the completeness of the mitochondrial genome assemblies for each lineage, see Supplemental Table S4. Mitochondrial genomes were annotated using MitoAnnotator (Iwasaki et al. 2013). Each of the 37 mitochondrial genes was aligned separately using MUSCLE (Edgar 2004). For ML phylogenetic reconstruction, we used IQ-TREE 2 (Minh et al. 2020) with 1000 bootstrap replicates.

### Demographic history

The PSMC model (Li and Durbin 2011) was employed to infer the history of the ancestral population dynamics of *Rhamphochromis*. PSMC reconstructed the historical changes in effective population size over time by analyzing the distribution of the most recent common ancestor between two alleles of an individual, with the parameter of “-u 3.5 × 10^–9^ -g 3” (Malinsky et al. 2018). For *Rhamphochromis* sp. “kingiri dwarf” and *Rhamphochromis* sp. “kingiri large,” additionally, the multiple sequential coalescent Markovian model (MSMC) method (Schiffels and Durbin 2014) was used to estimate historical changes in effective population sizes. The input files for MSMC were generated using msmc-tools (https://github.com/stschiff/msmc-tools). For each of the 14 individuals (six *Rhamphochromis* sp. “kingiri large” and eight *Rhamphochromis* sp. “kingiri dwarf”), only sites with uniquely mapped reads and sites with coverage depths ≥5× were used for the analyses. All sites were phased using Beagle v5.2 (Browning and Browning 2007). For effective population size inference, three individuals (six phased haplotypes) from each species were used. We also used the MSMC2 to calculate the relative cross-population coalescent rate (rCCR) with default parameter. rCCR of 0.5 was considered as an indicator of population split.

### GWA mapping

To investigate the genetic basis of body size variation across all *Rhamphochromis*, an association test was performed using PLINK v1.90 (Purcell et al. 2007). Briefly, the VCF file was first pruned to remove SNPs in strong linkage disequilibrium (*r*^2^ > 0.3) in every 2 kb window to keep only one SNP. Then PCA was performed for the pruned VCF file, and PC1 was extracted to represent the main axis of population genetic structure. Finally, an association test was calculated with the median value of length of body size (standard length) for each *Rhamphochromis* species as the response variable, as well as genomic PC1 as a covariate to account for population genetic structure.

### Association between SNPs and genes

We associated genes with SNPs from the GWA analysis if the SNPs were within the gene body or within ±5 kb from the transcription start and end sites. If no genic region overlapped the lead SNP, then we associated them with the closest genes within 10 kb.

### *f*-Branch statistics

To calculate excess allele-sharing across the data set and assess whether different species have evolved in the presence of interspecific gene flow, we computed the *f*-branch statistic *f*_b_(C) using Dsuite v.0.386 (Malinsky et al. 2021). First, we simplified the full RAxML phylogenetic tree with the R package ape (Paradis et al. 2004) to account for multiple individuals per species. We then applied Dsuite to calculate branch-specific estimates of gene flow, referred to as the “*f*-branch statistic.” Next, we calculated *f*_4_-ratios based on the relationships from the phylogenetic tree for all populations using the Dtrios program within Dsuite. The *f*-branch statistic for each phylogenetic branch was then estimated with the Fbranch program within Dsuite, using the parameter “-p 0.05.” Finally, we visualized the resulting *f*-branch statistics using the dtools.py script, again part of Dsuite.

### RNA sequencing data analysis

Whole-embryo bulk RNA sequencing data from early stages of *Rhamphochromis* sp. “chilingali” development were obtained from a recent study (Marconi et al. 2024). We first removed the adapter sequences and filtered the low-quality sequences (Phred < 20) using Trim Galore! v0.6.6 (https://github.com/FelixKrueger/TrimGalore). Then high-quality RNA-seq reads were mapped to the *A. calliptera* genome assembly (fAstCal1.2 in Ensembl 111), using STAR v.2.7.1a (Dobin et al. 2013) and only uniquely mapped reads retained for subsequent analysis. Finally, the number of reads mapped to each gene in the *A. calliptera* reference genome was counted in STAR using the built-in HTSeq-count option (Anders et al. 2015).

### Population genetics analysis

To compare estimates of genetic diversity of the two Lake Kingiri species, VCFtools v0.1.17 (Danecek et al. 2011) was used to calculate the average pairwise nucleotide diversity (π) and Tajima's D statistics within 50 kb sliding windows of 10 kb steps. To estimate the LD patterns within species, we calculated the mean *r*^2^-values for pairwise markers with a MAF greater than 0.05 using PopLDdecay (Zhang et al. 2019). These parameters were set to “-MaxDist 500 -MAF 0.05 -Het 0.88 -Miss 0.2.” The population scaled recombination rate (ρ = 4Ner) was calculated using FastEPRR (Gao et al. 2016).

### Genome-wide patterns of divergence for genomic islands

Genomic differentiation between the Kingiri fish pair was assessed using relative divergence (*F*_ST_) and absolute divergence (*D*_xy_) across the genome, calculated in 50 kb nonoverlapping windows. This analysis was performed using pixy v.1.2.11. beta1 (Korunes and Samuk 2021). To enable comparisons of genomic divergence landscapes across pairs with varying divergence times, per-window *F*_ST_-values were standardized to *Z*-transformed values (*ZF*_ST_) using the formula: *ZF*_ST_ = (*F*_ST_ – µ*F*_ST_) / σ*F*_ST_, where *F*_ST_ represents the window-specific value, µ*F*_ST_ is the mean *F*_ST_ across all windows, and σ*F*_ST_ is the standard deviation of *F*_ST_-values across the genome (Karlsson et al. 2007). Genomic regions with *ZF*_ST_ ≥ 4 were classified as genomic islands, with thresholds of *ZF*_ST_ ≥ 3.5 and *ZF*_ST_ ≥ 3 also being tested. Adjacent significant windows were merged into contiguous regions. The significance of *D*_xy_, π, and ρ within genomic islands was evaluated relative to the genomic background using the Wilcoxon rank-sum test.

### Identification of selected genomic regions

To identify the potential regions under selection for each of the Kingiri taxa, nucleotide diversity (π) and population fixation statistics (*F*_ST_) were calculated using VCFtools v.0.1.17 (Danecek et al. 2011) in a 50 kb sliding window with a step size of 10 kb. We filtered the window with the top 5% of *F*_ST_-values (*F*_ST_ ≥ 0.27) as the outlier windows. On this basis, log_2_ ratio values were used (log_2_ ratio of π-values: log_2_(π[large]/π[dwarf]) ≥ 0.39, top 5%; log_2_(π[GS]/π[GE]) ≤ –0.97, bottom 5%) to identify selected genomic regions in *Rhamphochromis* sp. “kingiri dwarf” and *Rhamphochromis* sp. “kingiri large,” respectively.

We also employed the integrated haplotype score (iHS) statistic (Voight et al. 2006) to detect genomic regions characterized by exceptionally long haplotypes in comparison to the rest of the genome to detect selective sweeps. Strongly negative iHS values signified increased haplotype homozygosity driven by a rise in the frequency of the derived allele, whereas positive values indicated a high frequency of the ancestral allele. These analyses were performed using the R package rehh v.3.2.2 (Gautier and Vitalis 2012).

We identified outlier *F*_ST_ windows across a 50 kb sliding window with a step size of 10 kb. We then identified genes overlapping with the outlier windows as outlier *F*_ST_-associated genes. We note this approach will identify genes under selection and also those genes in linkage disequilibrium with those genes under selection.

### GO enrichment analysis

We used the ortholog between *A. calliptera* and zebrafish to perform GO enrichment analysis, as zebrafish has the most extensive functional gene annotation. One-to-one orthologs between *A. calliptera* and zebrafish were downloaded from Ensembl (release 111) using BioMart database. In total, there were 15,158 one-to-one orthologs of the two species, from 28,001 *A. calliptera* genes and 37,241 zebrafish genes. The GO and KEGG enrichment analysis were carried out using the online tool DAVID (Sherman et al. 2022; https://david.ncifcrf.gov/) using the default statistical parameters. Significantly enriched GO terms were further imported to the online tool REVIGO to identify the most connected and represented GO terms (Supek et al. 2011).

## Data access

The sequence data generated in this study have been submitted to the NCBI BioProject database (https://www.ncbi.nlm.nih.gov/bioproject/) under accession number PRJNA1241418.

## Supplemental Material

Supplement 1

Supplement 2

Supplement 3

Supplement 4

Supplement 5

Supplement 6

Supplement 7

Supplement 8

Supplement 9

Supplement 10

Supplement 11

Supplement 12

Supplement 13

Supplement 14

Supplement 15

Supplement 16
