# Supplementary material for: Dynamic body size evolution during speciation of predatory cichlid fishes in the Lake Malawi superradiation
Source: Genome Res. 2026 May;36(5):949–63. doi: 10.1101/gr.281193.125 (PMC13445721; doi:10.1101/gr.281193.125)
Supplement: Supplementary file 1 [file Supplemental_Material.pdf]

Supplementary Materials

Yang et al. Dynamic body size evolution during speciation of predatory cichlid fishes in the Lake Malawi super-radiation

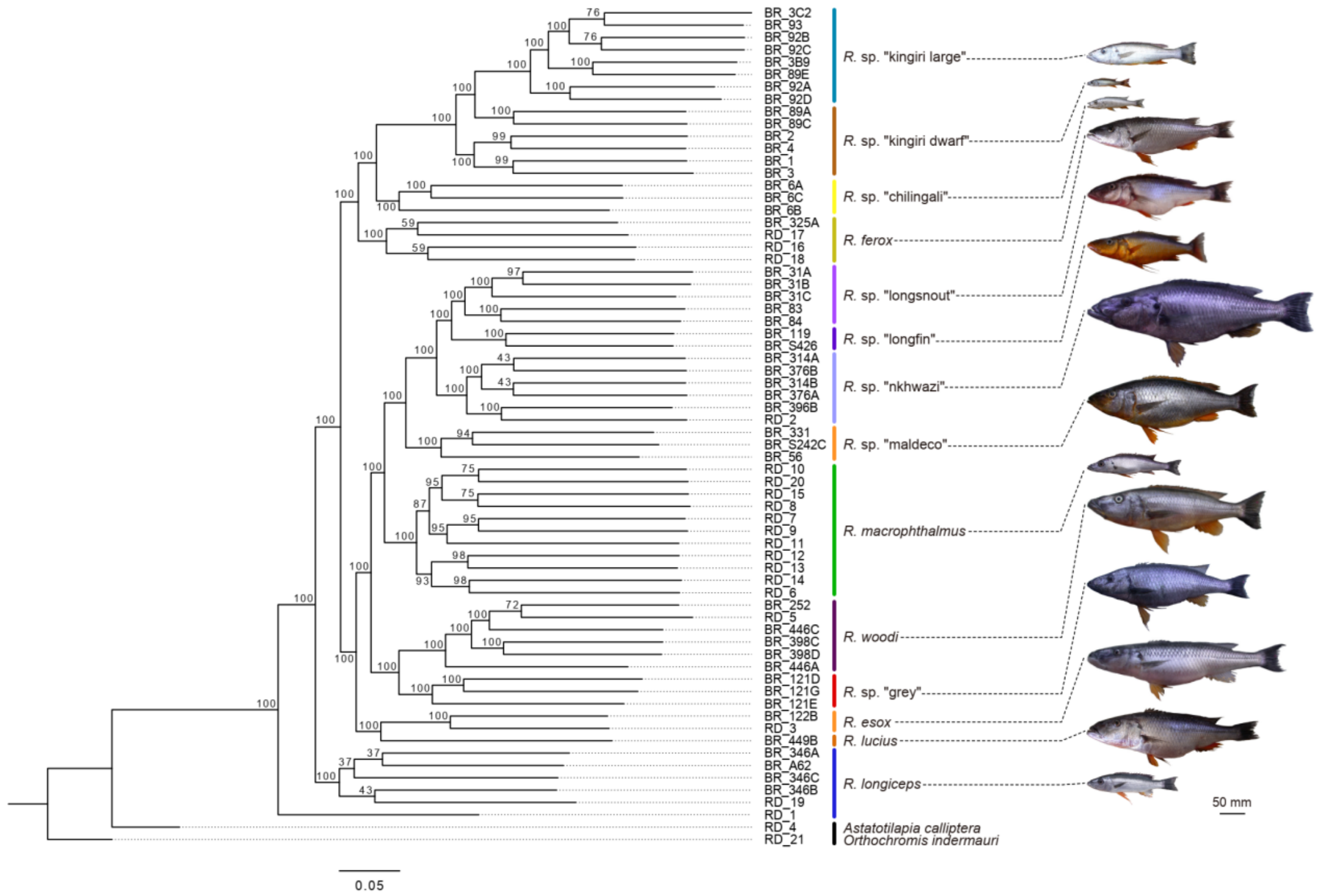

Supplemental_Fig_S1: Maximum likelihood phylogenetic tree constructed using whole-genome single-nucleotide polymorphism (SNP) data using IQTREE2 with 1000 bootstraps. The nodes separating each species have a bootstrap value of 100%.

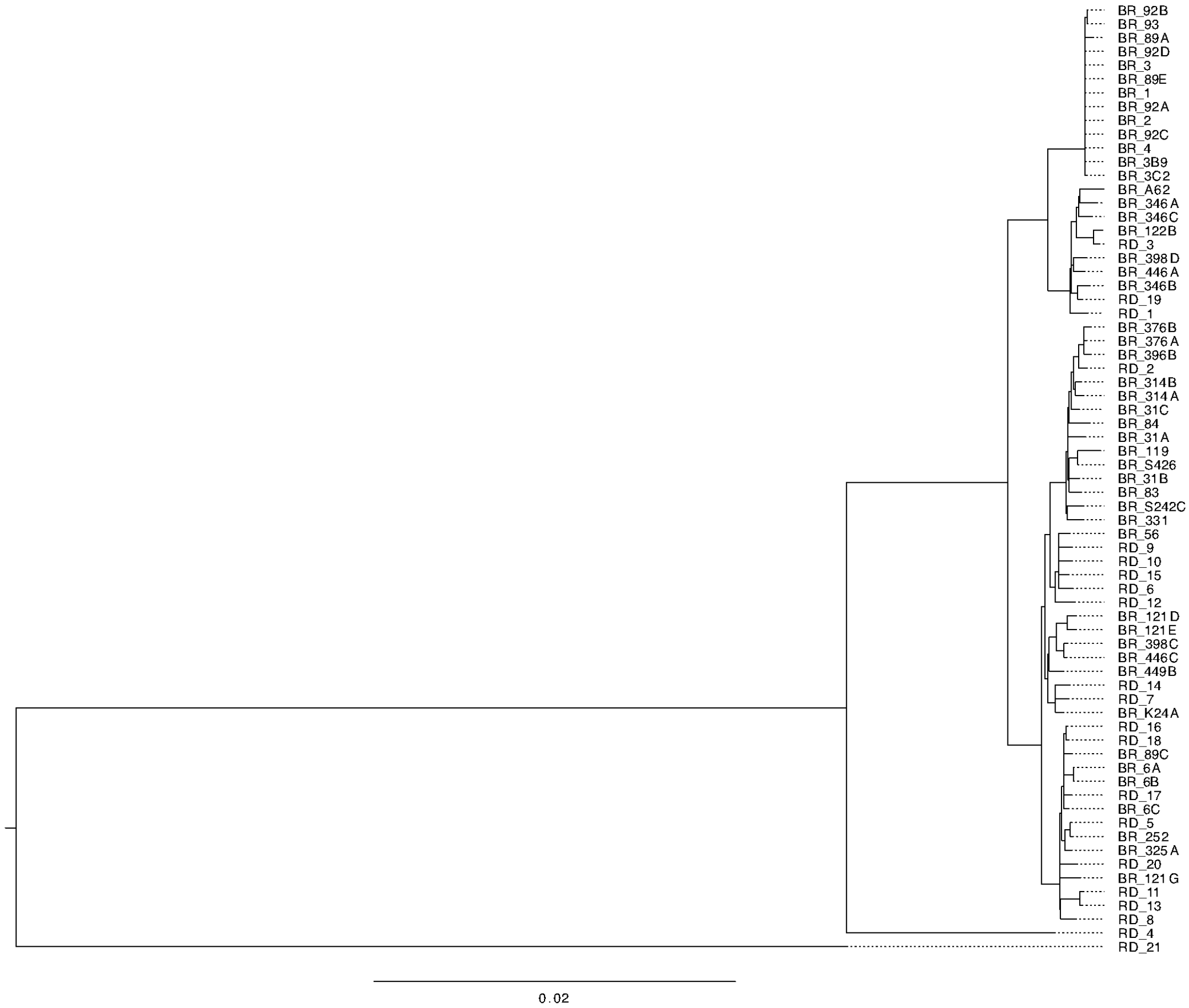

Supplemental_Fig_S2: Maximum likelihood phylogenetic tree constructed using 37 mitochondrial genes using IQTREE2 with 1000 bootstraps. Bootstrap values for each node are provided in Figure S3.

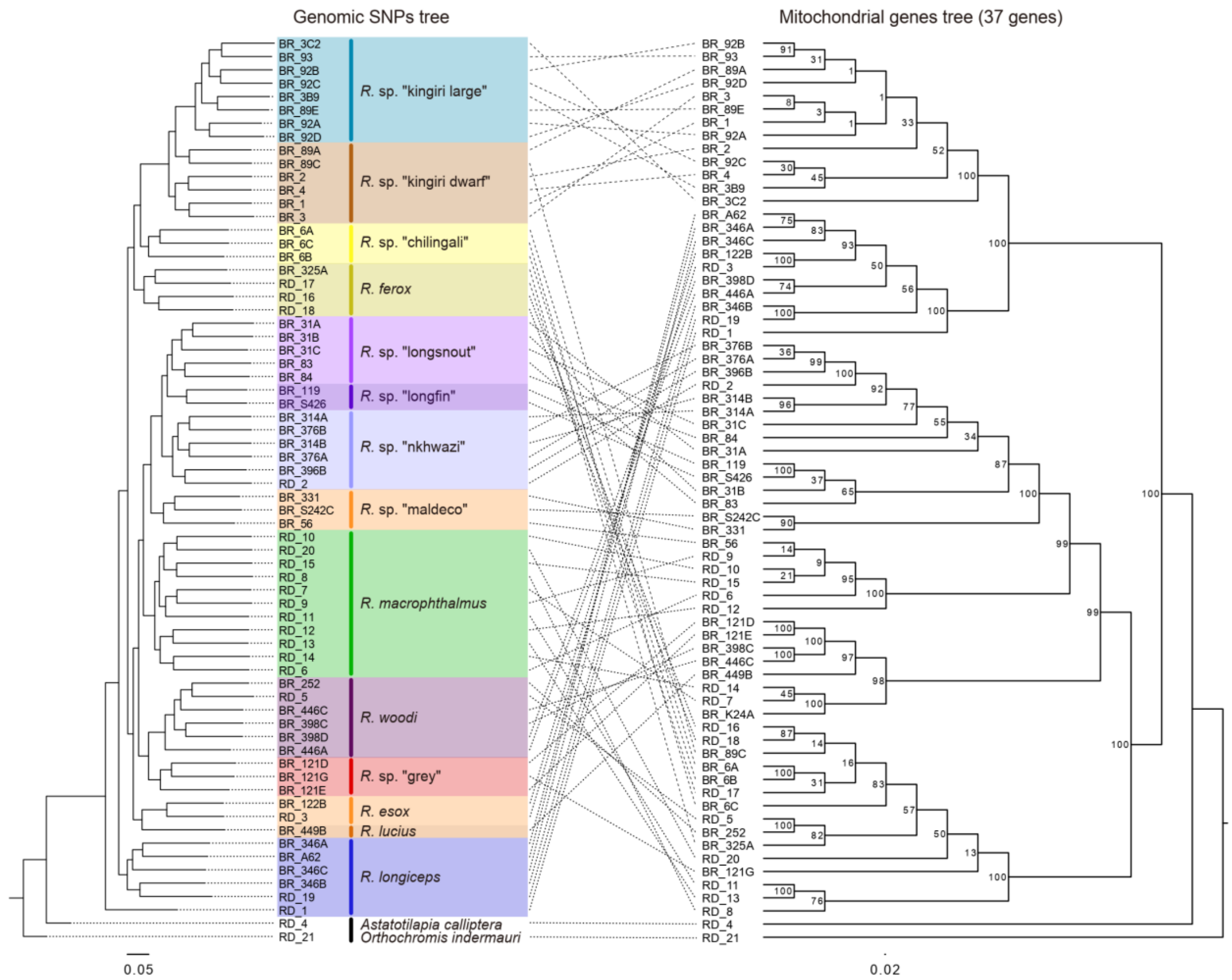

Supplemental_Fig_S3: Discrepancy between nuclear and mitochondrial trees. The discrepancies between phylogenies based on concatenated WGS SNP (left) and on mitochondrial genome data (right).

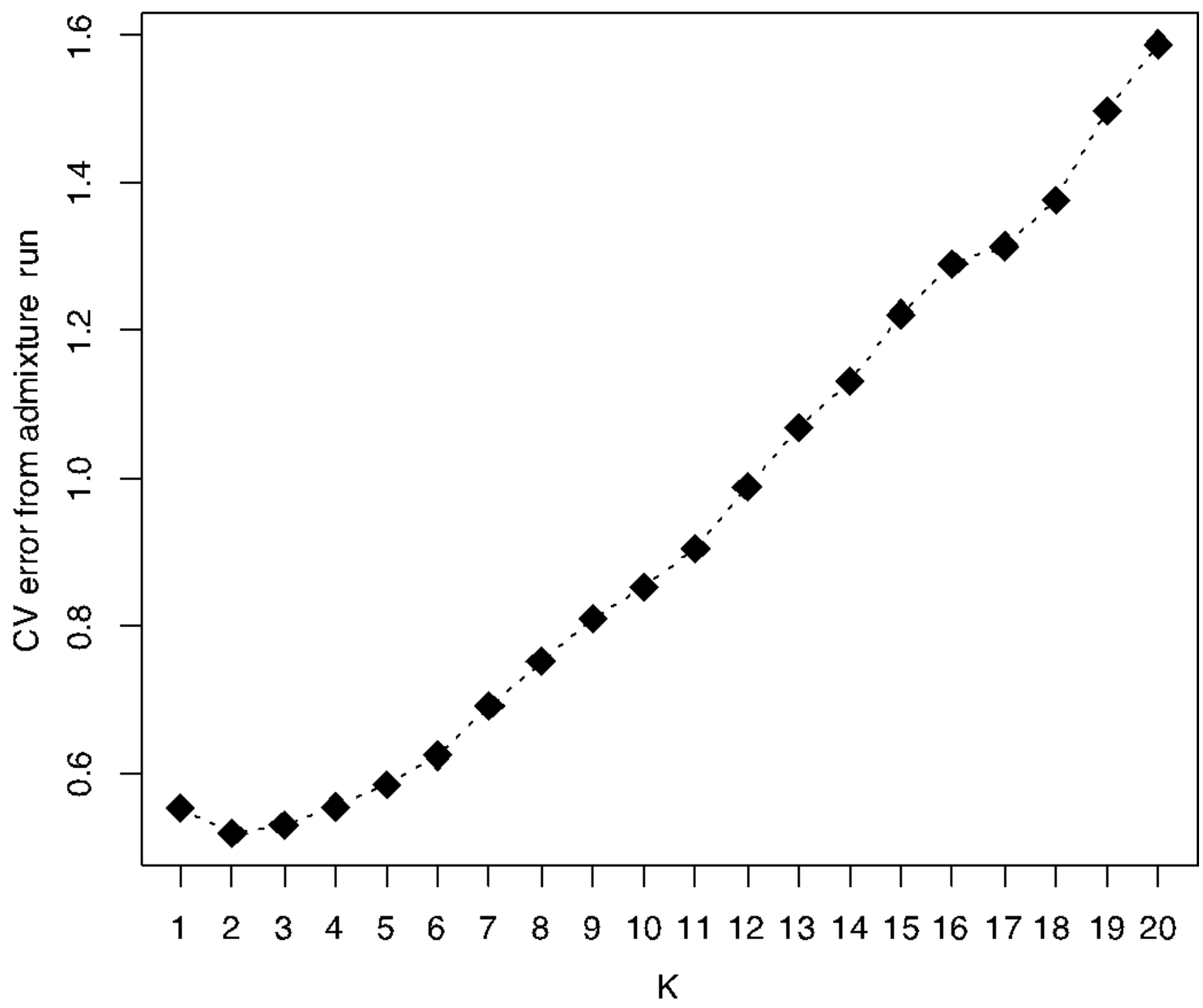

Supplemental_Fig_S4: CV error generally increased with the number of populations (K) for each admixture run, with K=2 having the lowest CV error. Lower values of K resulted in lower CV error, and higher values of K resulted in higher CV error. K=10 was selected for plotting since it helped identify species-specific differences.

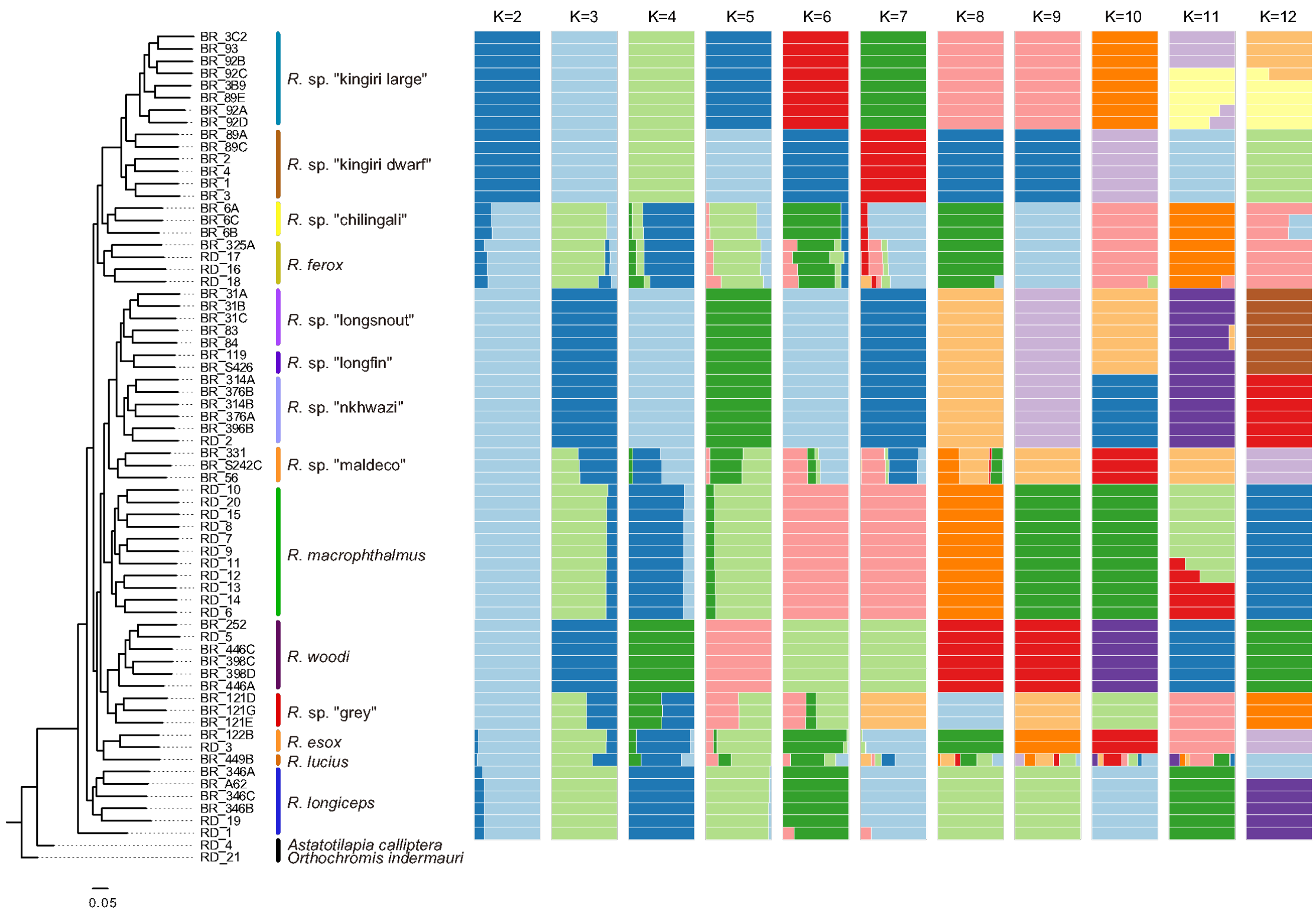

Supplemental_Fig_S5: Maximum likelihood IQTREE2 phylogeny (as Fig. 1) with admixture analysis where the number of populations (K) ranges from 2 to 12. Increased values of K resulted in more complex admixture patterns.

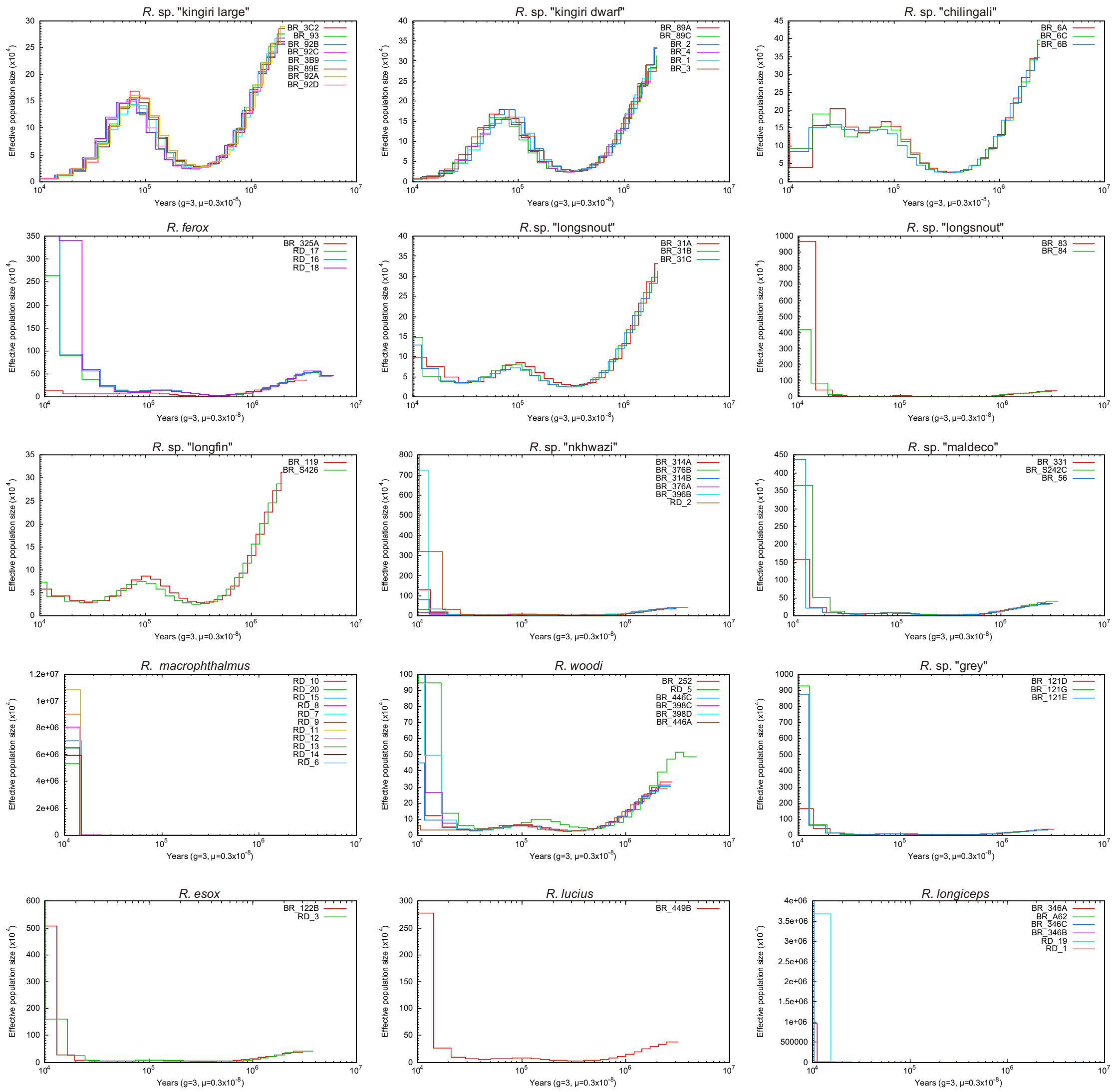

Supplemental_Fig_S6: Demographic history for each species inferred by PSMC. Many Lake Malawi species showed evidence of population expansion around 100,000 years ago. Generation time (g) is 3 years and mutation rate (μ) of 3.5×10^{-9} per site per generation.

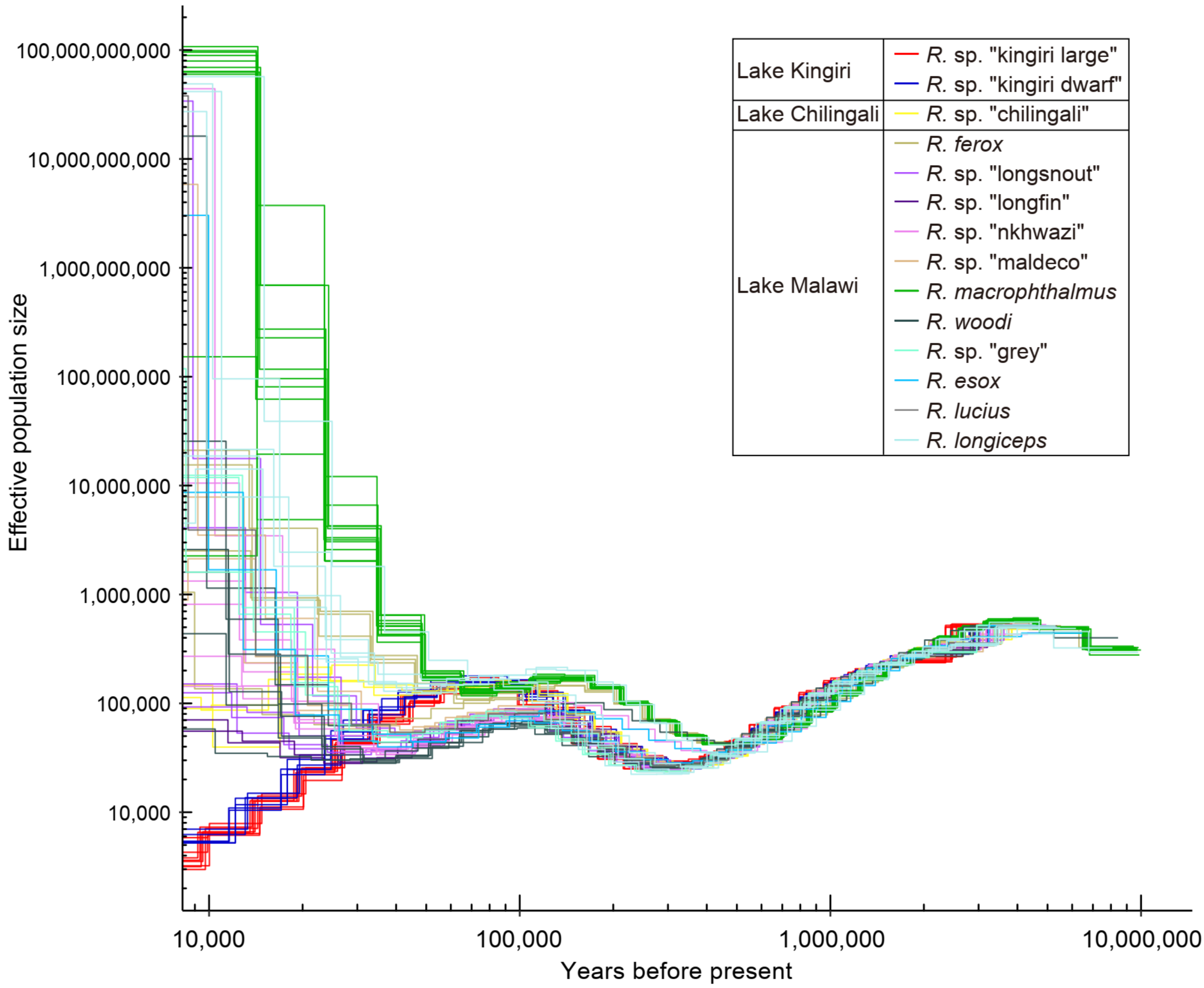

Supplemental_Fig_S7: Demographic history for each species inferred by PSMC. Many Lake Malawi species showed evidence of population expansion around 100,000 years ago. Generation time (g) is 3 years and mutation rate (μ) of 3.5×10^{-9} per site per generation.

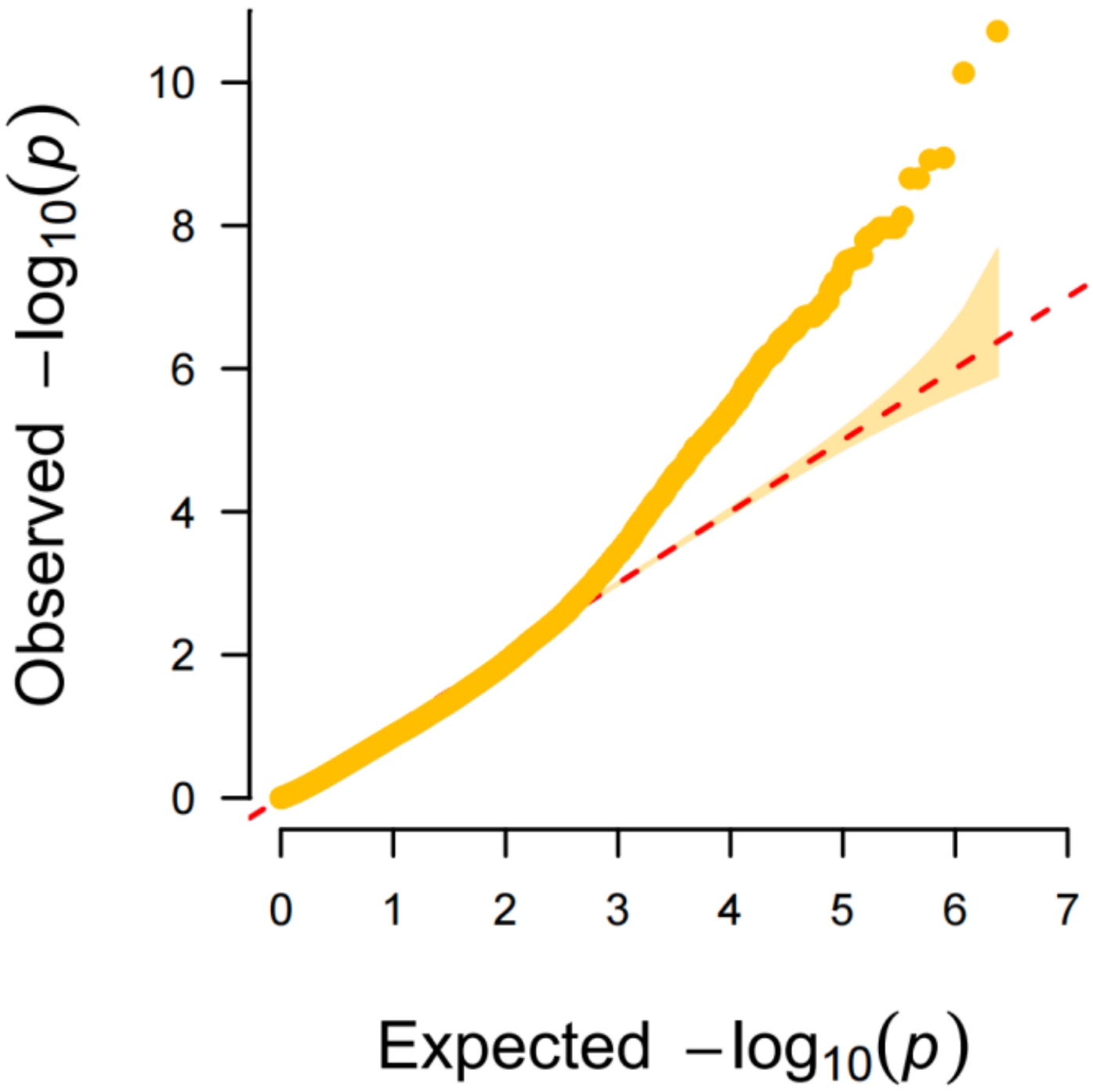

Supplemental_Fig_S8: Q-Q plot showing the expected -$\log_{10}$(*P*-values) vs. the observed -$\log_{10}$(*P*-values) for the GWA analysis of body size evolution across all 66 *Rhamphochromis* individuals

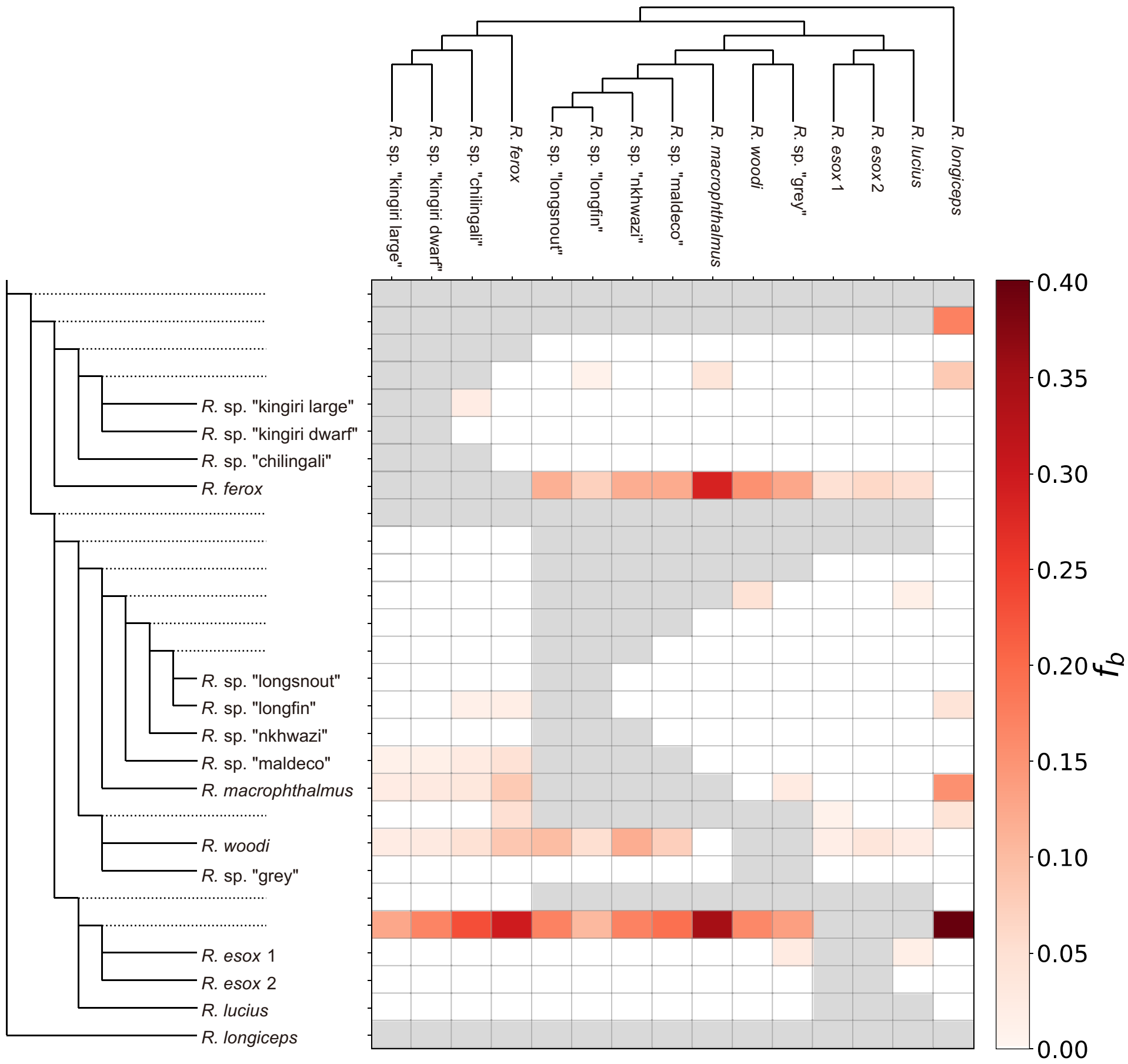

Supplemental_Fig_S9: Heatmap showing the estimated admixture fractions between pairs of species represented on the x- and y-axes. Species are arranged along the axes according to their placement in the species tree. The matrix values (f_b) were calculated using Dsuite's *f*-branch statistic, which quantifies excess allele sharing between the branch specified on the expanded tree along the y-axis (relative to its sister branch) and the species identified on the x-axis. These values provide insights into the hybrid origin of shared allele. Here, two individuals of *R. esox* were separated to show the admixture was in the ancestor of *R. esox*.

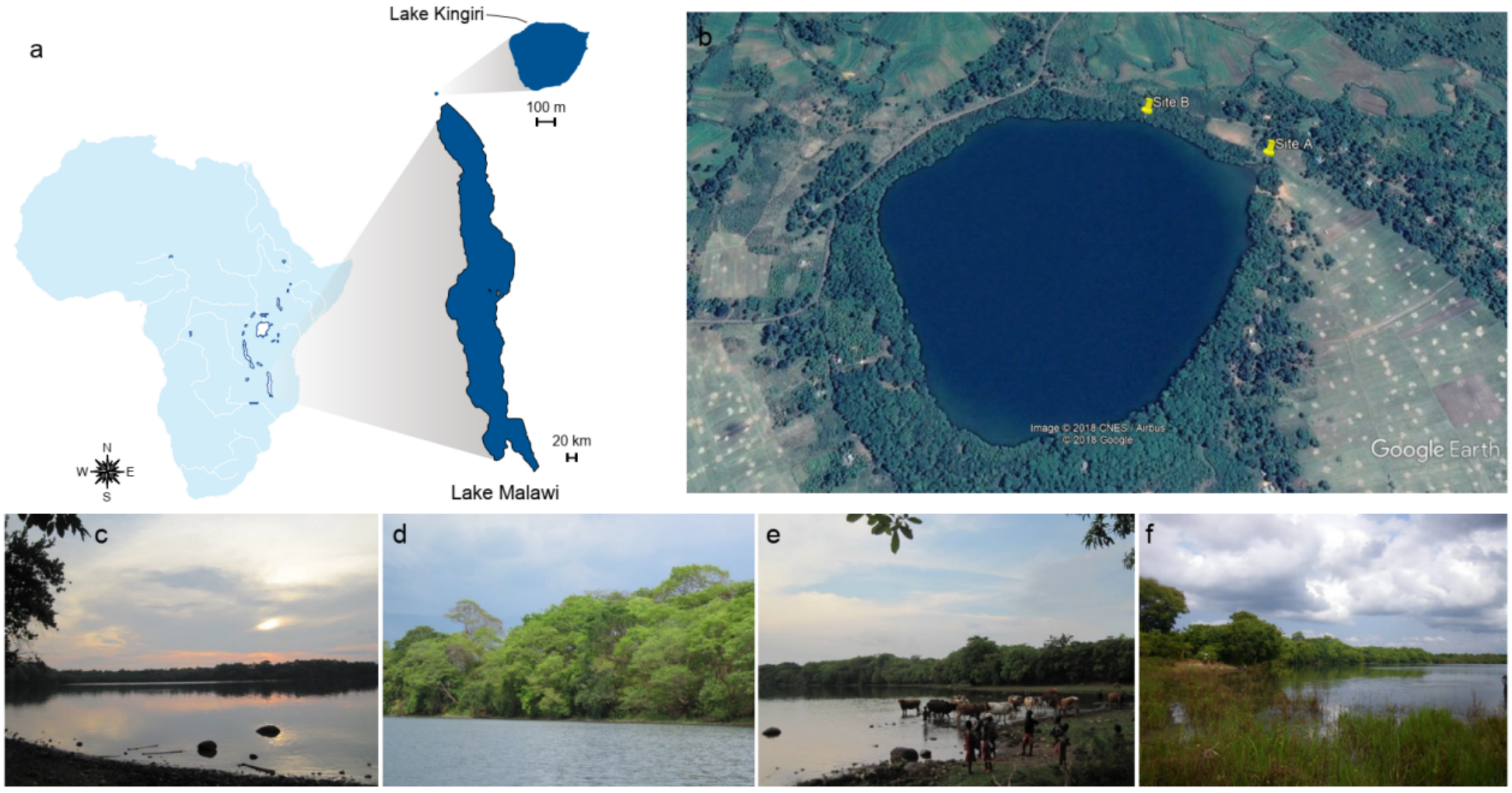

Supplemental_Fig_S10: Lake Kingiri (a-b), showing main access points used in surveys: Site A (b) is a shallow open area with seasonally submerged grasses, used for washing and livestock; Site B (b) marks the approximate location of several narrow paths through dense woodland, leading to a rapid drop-off underwater. (c-f) Panoramic view from Site A, looking roughly SW (c); heavily wooded shoreline in the vicinity of site B (d), viewed from a boat on the lake; site A, showing heavy use for watering livestock (e); site A, flooded emergent grasses in the foreground with open beach used for drying clothes behind (f).

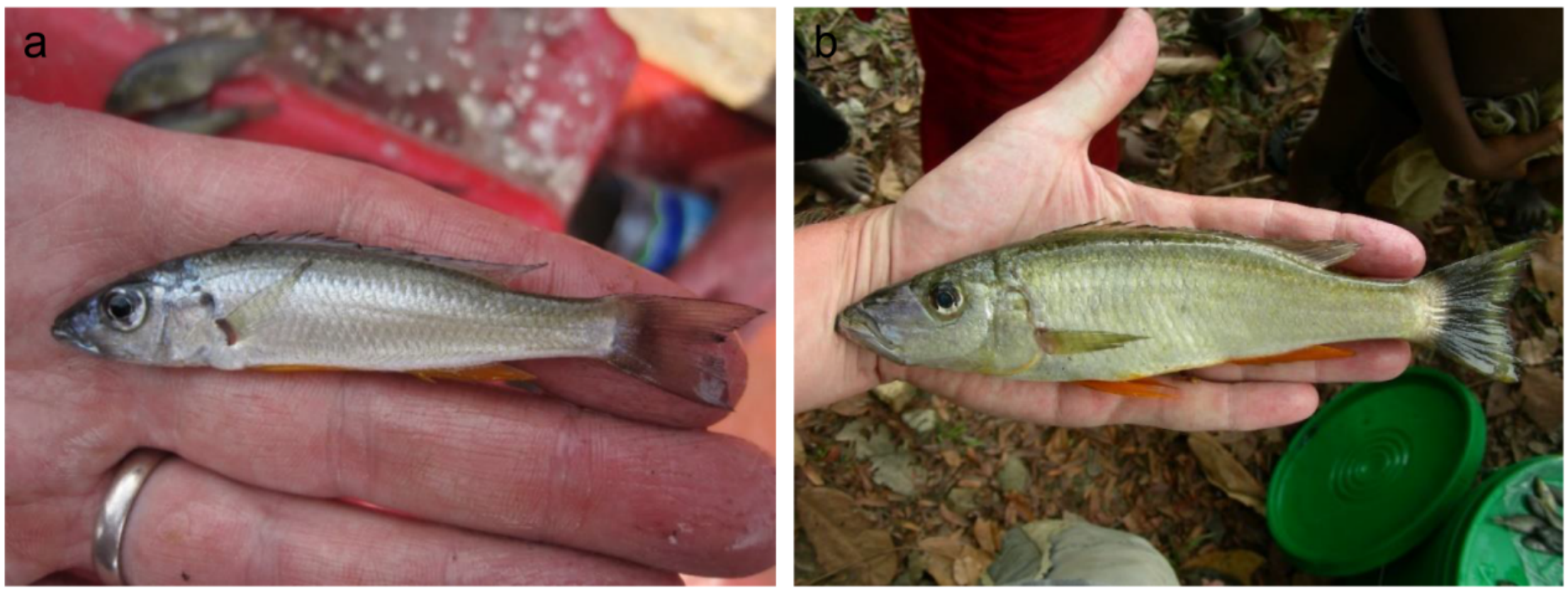

Supplemental_Fig_S11: Photographs of *Rhamphochromis* sp. "kingiri dwarf" (a) and *Rhamphochromis* sp. "kingiri large" (b) in Lake Kingiri, showing substantial body size differences in mature males, as indicated by the bright orange pelvic and anal fins.

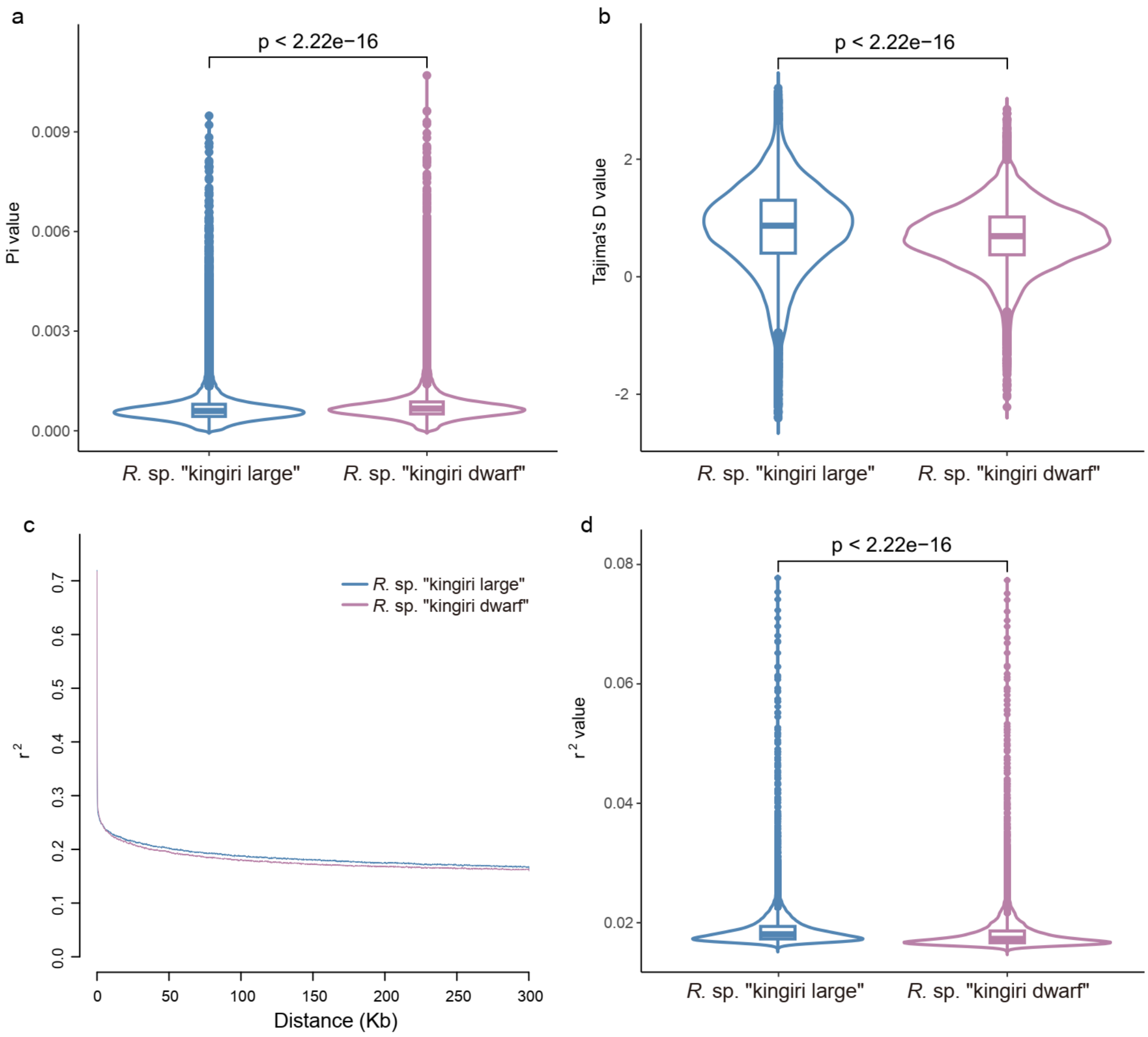

Supplemental_Fig_S12: Genome wide nucleotide diversity (π) (a), Tajima's *D* (b), LD decay patterns (c), and linked disequilibrium (LD) values (r^2) (d) of *R. sp*. "kingiri large" and *R. sp*. "kingiri dwarf".

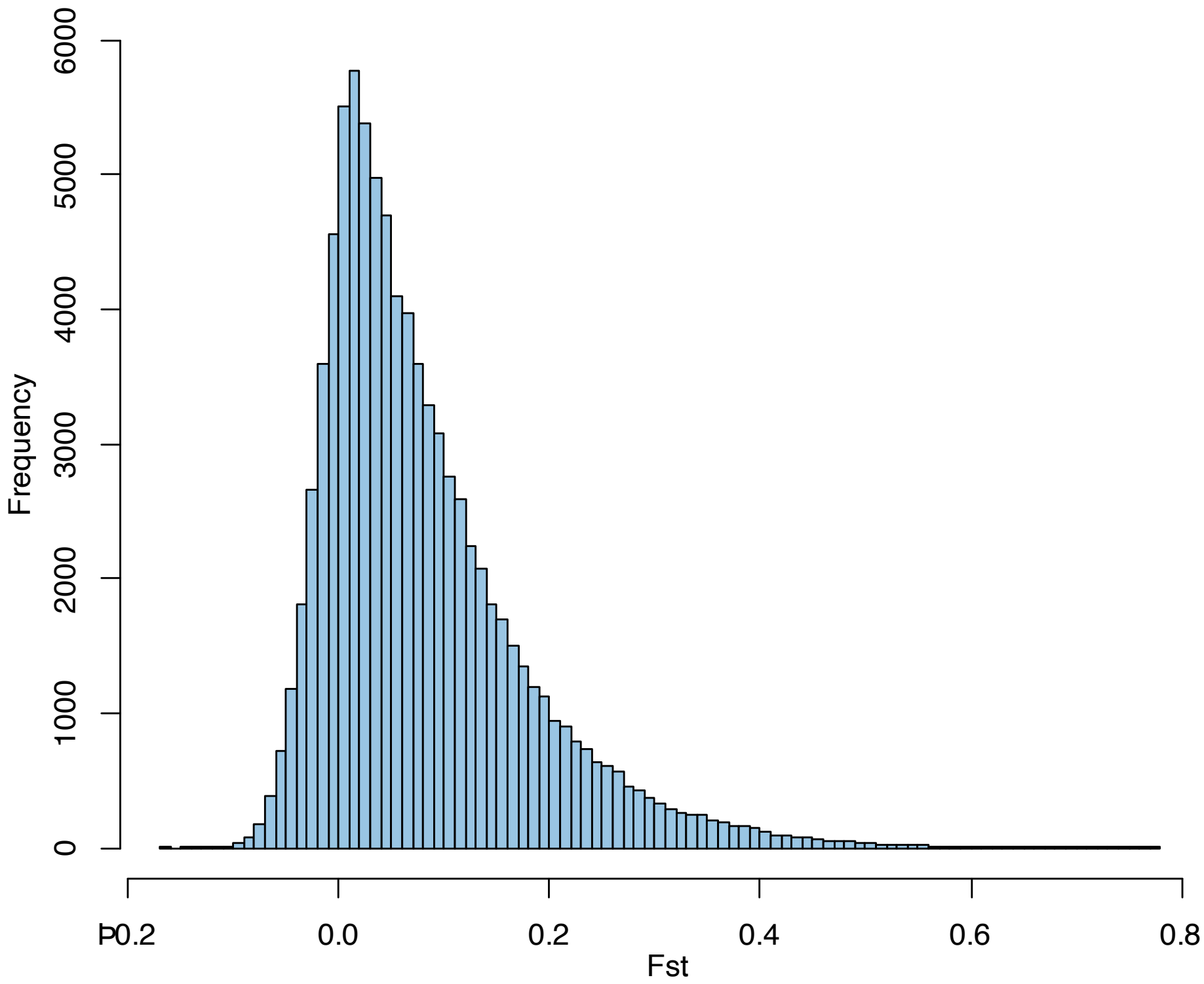

Supplemental_Fig_S13: Distributions of F_{ST} measured in 50 kb windows across the genome for a comparison of *R.* sp. "kingiri large" and *R.* sp. "kingiri dwarf".

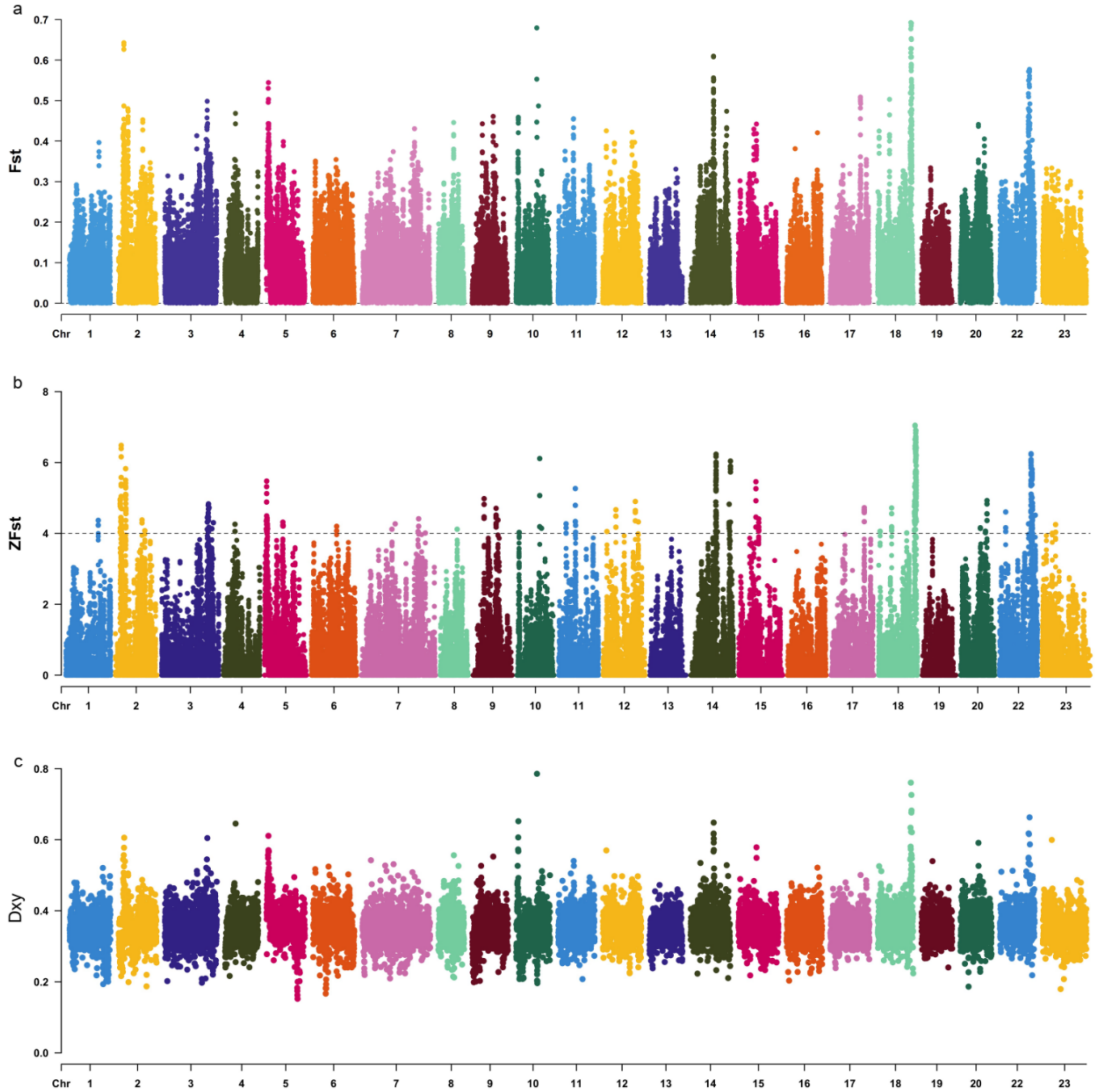

Supplemental_Fig_S14: Genome-wide pattern of F_{ST} (a), ZF_{ST} (b), and Dxy (c) divergence measured in 50-kb non-overlapping windows for *R.* sp. "kingiri large" and *R*. sp. "kingiri dwarf".

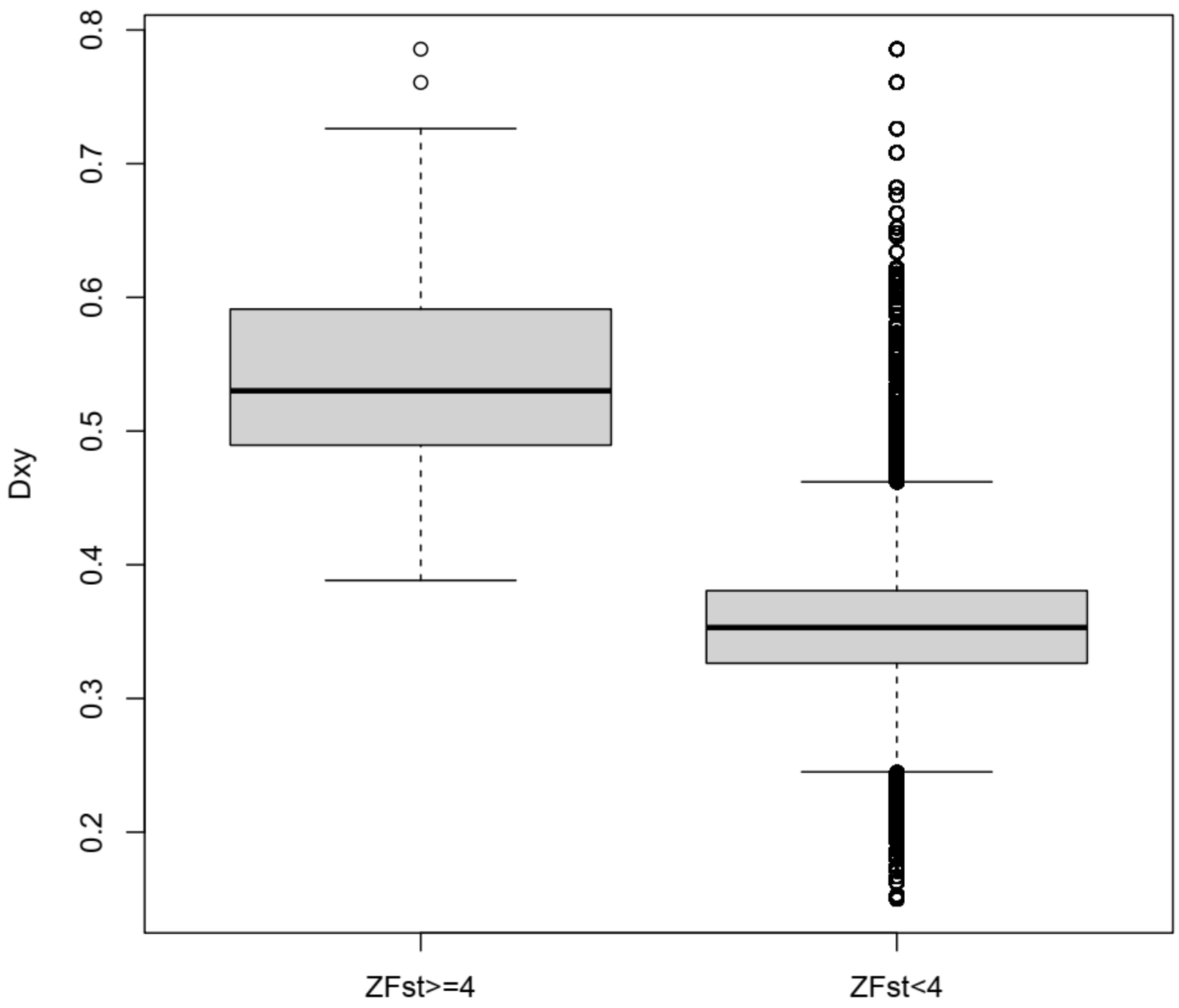

Supplemental_Fig_S15: Absolute measures of divergence (Dxy) between genomic islands of divergence (ZF_{ST}>=4) and the genomic background (ZF_{ST}<4), for *R*. sp. "kingiri large" and *R*. sp. "kingiri dwarf".

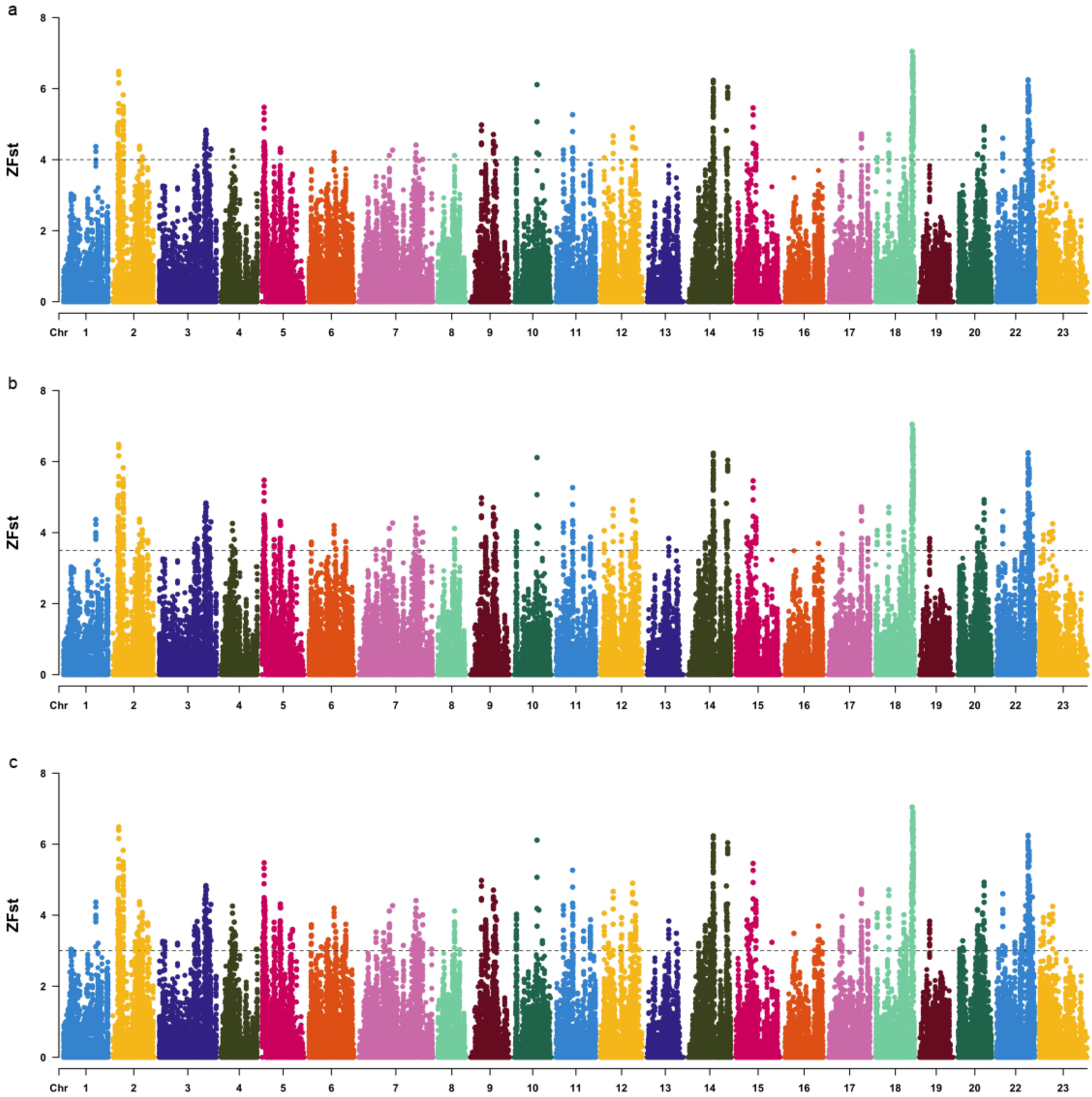

Supplemental_Fig_S16: Genome-wide pattern of ZF_{ST} divergence with cutoff was set at 4 (a), 3.5 (b), and 3 (c) measured in 50-kb non-overlapping windows for *R*. sp. "kingiri large" and *R*. sp. "kingiri dwarf".

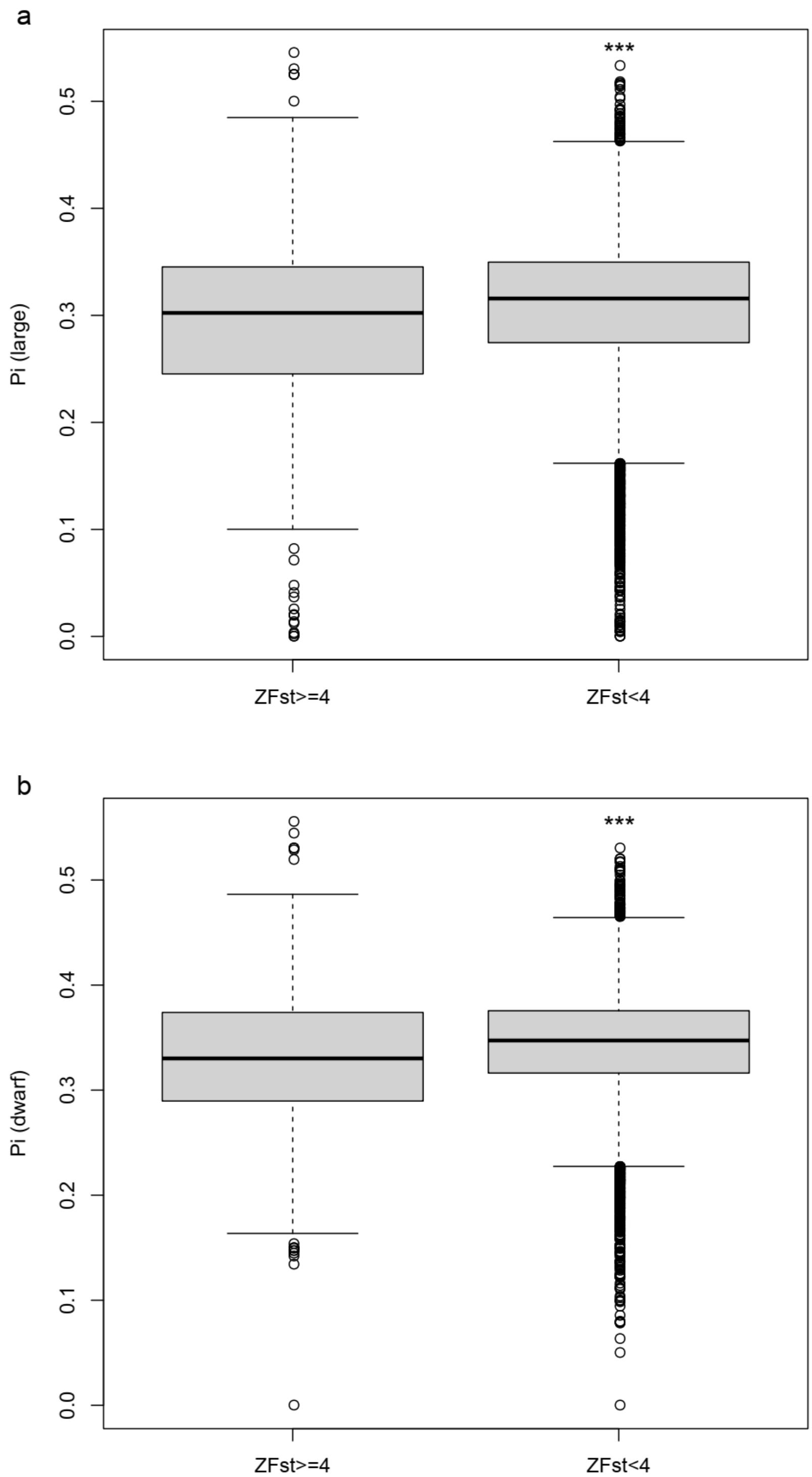

Supplemental_Fig_S17: Comparison of nucleotide diversity (π) measured between genomic islands (ZF_{ST} >=4) and the genomic background (ZF_{ST} <4) for *R.* sp. "kingiri large" (a) and *R.* sp. "kingiri dwarf" (b). Significant differences of Mann–Whitney U test are indicated with *** $P < 1.5\times10^{-5}$.

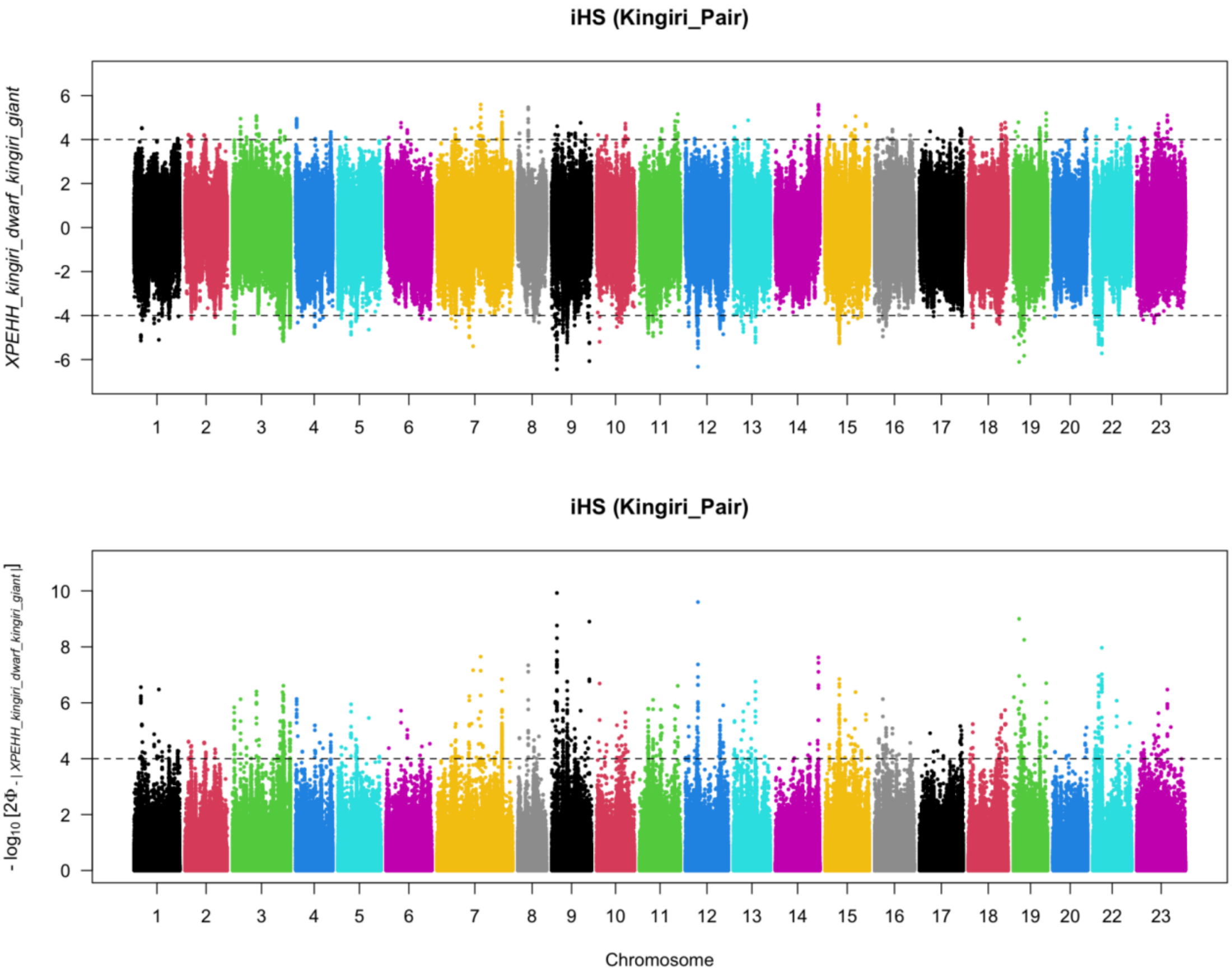

Supplemental_Fig_S18: Selective sweeps detected using rehh from the genome-wide comparison of *R*. sp. "kingiri large" and *R.* sp. "kingiri dwarf".
